# Hepatocarcinogenesis Prevention by Pirfenidone Is PPARγ Mediated and Involves Modification of Nuclear NF-kB p65/p50 Ratio

**DOI:** 10.3390/ijms222111360

**Published:** 2021-10-21

**Authors:** Jorge Antonio Silva-Gomez, Marina Galicia-Moreno, Ana Sandoval-Rodriguez, Hipolito Otoniel Miranda-Roblero, Silvia Lucano-Landeros, Arturo Santos, Hugo Christian Monroy-Ramirez, Juan Armendariz-Borunda

**Affiliations:** 1 Centro Universitario de Ciencias de la Salud, Instituto de Biologia Molecular en Medicina, Universidad de Guadalajara, Guadalajara 44340, Mexico; xntonio.silva@gmail.com (J.A.S.-G.); marina.galicia@academicos.udg.mx (M.G.-M.); anasol44@hotmail.com (A.S.-R.); mroblero7@hotmail.com (H.O.M.-R.); silvia.lucano@gmail.com (S.L.-L.); 2 Tecnologico de Monterrey, Escuela de Medicina y Ciencias de la Salud, Zapopan 45138, Mexico; arturo.santos@tec.mx

**Keywords:** HCC, PPARγ, inflammation, apoptosis, liver cancer

## Abstract

Targeted therapies for regulating processes such as inflammation, apoptosis, and fibrogenesis might modulate human HCC development. Pirfenidone (PFD) has shown anti-fibrotic and anti-inflammatory functions in both clinical and experimental studies. The aim of this study was to evaluate PPARγ expression and localization in samples of primary human tumors and assess PFD-effect in early phases of hepatocarcinogenic process. Human HCC tissue samples were obtained by surgical resection. Experimental hepatocarcinogenesis was induced in male Fischer-344 rats. TGF-β1 and α-SMA expression was evaluated as fibrosis markers. NF-kB cascade, TNFα, IL-6, and COX-2 expression and localization were evaluated as inflammation indicators. Caspase-3, p53, and PARP-1 were used as apoptosis markers, PCNA for proliferation. Finally, PPARα and PPARγ expression were evaluated to understand the effect of PFD on the activation of such pathways. PPARγ expression was predominantly localized in cytoplasm in human HCC tissue. PFD was effective to prevent histopathological damage and TGF-β1 and α-SMA overexpression in the experimental model. Anti-inflammatory effects of PFD correlate with diminished IKK and decrease in both IkB-phosphorylation/NF-kB p65 expression and p65-translocation into the nucleus. Pro-apoptotic PFD-induced effects are related with p53 expression, Caspase-3 p17 activation, and PARP-1-cleavage. In conclusion, PFD acts as a tumor suppressor by preventing fibrosis, reducing inflammation, and promoting apoptosis in MRHM.

## 1. Introduction

Hepatocellular carcinoma (HCC) is the main primary liver neoplasm worldwide and is the fourth most common cause of cancer-related death and the sixth in terms of incidence. According to data from the World Health Organization, more than 1 million patients will die of liver cancer in 2030 [1]. HCC pathophysiology includes clinical events of chronic liver disease such as products of sustained inflammation, fibrosis, and aberrant hepatocyte regeneration. These abnormal events allow genetic changes in key signaling pathways that culminate in dysplastic nodule formation and cancer [1]. Etiological factors include those that are highly inflammation-inducers such as hepatitis B virus, hepatitis C virus, diabetes, obesity, excessive alcohol intake, and metabolic diseases, which contribute to fibrosis/cirrhosis and HCC development [2]. In the premalignant stage, dysregulated cytokine production (IL-1-β, IL-6, TNFα), macrophage, and myeloid cell infiltration favor proinflammatory chronic pathway activation such as those related to nuclear factor kappa-B (NF-kB) [2]. NF-kB is a pleotropic transcription factor that regulates the expression of genes that promotes cell growth, survival, and neoplastic transformation in some tumors [3,4]. In its canonical signaling pathway, NF-kB, is a heterodimer composed of p50 and p65/RelA subunits. In unstimulated cells, this heterodimer is sequestered in the cytoplasm by p65-bound IkB-α; upon stimulation, IkB-α is phosphorylated by IKK-α and targeted for proteasome degradation. The release of NF-kB p65/p50 promotes its nuclear translocation, favoring the transcription of target genes that influence cell proliferation, migration, and inflammation, essential processes in cancer development [4]. The peroxisome proliferator-activated receptor (PPAR) family, such as PPARα and PPARγ, also play an anti-inflammatory role in liver injury. Generally, genes coding for these proteins are involved in lipid oxidation, however they also have properties that are associated with gene expression [5]. PPARγ has recently been considered as a possible therapeutic target in the treatment of different cancers. Stimulation of PPARγ can inhibit neoplastic processes by suppressing tumor cell replication and decreased survival of tumor cells [6]. When PPARα is bound to NF-kB p65, it inactivates its translocation to the nucleus and reduces the proinflammatory response [7,8]. In addition, PPARγ promotes NF-kB ubiquitination, which leads to its degradation, and therefore to its ability to generate an anti-inflammatory effect [9,10]. In addition, biochemical interaction studies showed that PPARγ interacts with p53 in a dependent manner, suggesting that PPARγ noncanonical ligands modulate p53 intrinsic activity [11].

Diagnosis of HCC is carried out in late stages mostly. Therefore, the use of animal models represents an adequate strategy for the evaluation of new pharmacological therapies in early stages. MRHM is a hepatocarcinogenesis model of chemical damage, which uses diethylnitrosamine (DEN), 2-acetylaminofluorene (2-AAF), and partial hepatectomy (PH) to alter hepatocytes and simulate the initiation, promotion, and progression events in HCC development [12]. DEN acts as an initiating agent by causing base modifications, and DNA breaks, while 2-AAF functions as a damage promoter by reducing the growth of normal hepatocytes, and PH triggers the growth of hepatocytes already modified by exposure to DEN. Treatment basically leads to clonal expansion of modified cells into foci of altered hepatocytes (FAHs), which can become nodules. Additionally, both DEN and 2-AAF can cause liver cholestasis and fibrosis [12,13,14].

On the other hand, pirfenidone (5-methyl-1-phenyl-2-(1H)-pyridone; PFD) is a small molecule with well-documented anti-fibrotic, antioxidant and anti-inflammatory properties [15,16]. PFD has shown changes in cytokines and growth factors expression, such as TGF-b1, PDGF, INF-y, TNF-a, IL-1b, IL-6, IL8 in the lungs; TGF-b1 and MMP9 in the heart; mRNA of TGF-b1 in the kidney; and TGF-b1, MMP2 and TMP-1 in the liver in several animal models [17]. Furthermore, this drug was able to decrease significantly infiltrating macrophages, specially M1 phenotype in a nephrectomized rat model [18], which was associated with a reduced hepatic T-cell and macrophage recruitment, as well as with the induction of M2-dominant polarized macrophages/Kupffer cells [19].

Furthermore, in vitro model reports have shown that PFD inhibits proliferation and promotes apoptosis of HCC cells and non-small cell lung cancer [20,21]. Recently we have demonstrated that PFD is an agonist ligand for PPARα with beneficial effects in NASH prevention in an experimental model [22]. In this study, our aim was to elucidate the mechanisms underlying PFD administration in the early stages of HCC development by using MRHM.

## 2. Materials and Methods

### 2.1. Animals

All animals received human care according to the Guide for the Care and Use of Laboratory Animals. Male Fischer-344 rats were provided by UPAE-Bioterio at CUCS, University of Guadalajara, Mexico, carried out in accordance with the guidelines of the University of Guadalajara under the approval number of the bioethics and research committees CI-03020, and additionally, in accordance with the ARRIVE guidelines. All animals were kept at 25 ± 2 °C with 12-h light/dark cycles with food and water ad libitum.

### 2.2. MRHM Design

Male Fischer-344 rats weighing 180 g, were distributed in three groups: non-treated (NT) (*n* = 10), complete treatment for induction of HCC group (CT) (*n* = 10) and complete treatment plus PFD administration for thirty days (CT/PFD30) (*n* = 10). NT group was used as sham operated group. As a first step, an intraperitoneal single dose of DEN (200 mg/kg, Sigma Aldrich, St. Louis, MO, USA) was administered to initiate a carcinogenic process marked as day zero. Subsequently, intragastric doses of 2-AAF (20 mg/kg, Sigma Aldrich) were administered consecutively at days seven, eight and nine. Finally, a PH was carried out (70% of the liver was removed; RML, right median lobe; LML, left median lobe; LLL, left lateral lobe) to induce a proliferative stimulus. CT group was administered simultaneously with 0.5% carboxymethyl cellulose orally (CMC 0.5%), and CT/PFD30 group was administered with PFD 500 mg/kg (donated by Cell Pharma S.A. de C.V., Jiutepec, Mexico), suspended in CMC 0.5%, daily from day zero until sacrifice (day 30) Macroscopic images were captured with Nikon D5500 camera.

### 2.3. Human Tissue Samples

Ten HCC tissue samples and three normal liver tissues were obtained by surgery and donated by the Hospital Civil de Guadalajara for further analysis, see Table S1 in Supplementary Material. Tumor grade was determined by two independent pathologists. AJCC Stage; IA, a single tumor 2 cm (4/5 inch) or smaller that has not grown into blood vessels (T1a) and not spread to nearby lymph nodes (N0) or to distant sites (M0).

### 2.4. Liver Histological Assessment

Liver samples (RLL, right lateral lobe) from all animals were fixed with 10% formaldehyde for 24 h. Tissues were washed, dehydrated in alcohol, and embedded in paraffin. Sections of 4 μm samples were mounted on glass slides. All samples were stained with Hematoxylin Eosin (H&E) and Masson’s according to standard procedures.

### 2.5. Protein Extraction and Western Blot

Liver tissue (RLL, 100 mg) and cells (2 × 10^6^) were treated as described in the supplementary information. Proteins were electro-transferred to PVDF membranes. Membranes were then incubated at 4 °C overnight with the corresponding primary antibody (Table S3 in Supplementary Material) and with secondary antibody (Anti-Mouse/anti-Rabbit IgG-POD, BM Chemiluminescence Western Blotting Kit Mouse/Rabbit, Roche, Mannheim, Germany). Bands of interest were visualized using BioRadChemiDoc™ XRS+ System software (Bio-Rad, Hercules, CA, USA).

### 2.6. Cell Culture

HepG2 cells were obtained from ATCC (HB-8065; 41106514, H87), and grown in DMEM medium supplemented with 10% (*v*/*v*) fetal bovine serum (Invitrogen Life Technologies-GIBCO, Carlsbad, CA, USA), 100 U/mL penicillin, 100 g/mL streptomycin, and maintained under humidified atmosphere of 5% CO_2_ at 37 °C. Cells were incubated with 500 μM PFD, 1 μM GW7647 (PPARγ agonist; Santa Cruz Biotechnology, Santa Cruz, CA, USA), and 100 nM GW9662 (PPARγ antagonist; Santa Cruz Biotechnology) for 24 h to evaluate PFD effect on PPARα and PPARγ proteins expression. Incubation was performed after 8 h of fetal bovine serum starving.

### 2.7. Immunofluorescence of Human and Rat Tissues and Cells

Immunofluorescence (IF) of human and rat tissues, in addition to HepG2 cells (1 × 10^3^), is described in detail in supplementary information. Liver tissues and cells were analyzed by confocal microscopy using a ZEISS laser scanning microscope LSM 800. Maximum projection and fluorescence intensity were analyzed with the free access software ZEN 2.3 SP1 (Carl Zeiss Microscopy, Oberkochen, Germany).

### 2.8. Molecular Docking and Structure Viewing

SwissDock web service molecular docking that predicts molecular interactions and powered by the software EADock DSS was used (SIB, Swiss Institute of Bioinformatics, Basel, Switzerland) [23]. UCSF CHIMERA software to visualize the molecular structure and predicted binding modes was used. Crystal structure of the human PPARγ-ligand binding domain (LBD) was obtained from the Protein Data Bank (PBD) with accession code PBD: 6O67. Rosiglitazone (ZINC968328) and PFD (ZINC1958) 3D structures were obtained from the ZINC database. Site direct mutations in PPARγ amino acids binding to PFD were performed using Swiss-Model Server.

### 2.9. Statistical Analysis

Density bands are expressed as the mean values ± SD. Two-tailed Student’s t-test or ANOVA followed by Tukey’s was used to test statistical significance between groups as appropriate. Pearson’s correlation was performed for colocalization microscopic analysis. Graphs and statistical analysis were generated with the Graph PadPrism7 software (GraphPad Software, San Diego, CA, USA). Differences were considered statistically significant when *p* < 0.05.

For further details regarding the materials used, please refer to Supplementary Materials.

## 3. Results

### 3.1. PPARγ Is Overexpressed in the Cytoplasm of Hepatocytes in Human HCC Tissue

**Figure 1:**
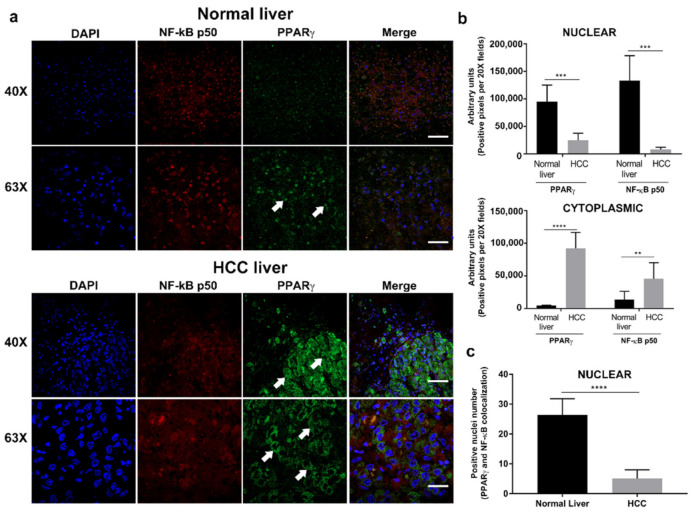
PPARγ and NF-kB p50 differential expression in human tissues. (**a**) Double IF of PPARγ and NF-kB p50 in normal liver tissue and HCC. (**b**) Representative chart of IF quantification in both tissues. (**c**) Nuclei positive for PPARγ and NF-kB p50 quantified in different fields of normal and HCC liver. Values are presented as means ± SD. HCC: hepatocellular carcinoma. ** *p* < 0.01, *** *p* < 0.001, **** *p* < 0.0001.

Expression and localization of PPARγ and NF-kBp50 were examined by double IF. Figure 1a (upper panel) shows the expression and co-localization of NF-kBp50 and PPARγ in the nucleus of hepatocytes of normal human liver tissue. In human HCC tissue, NF-kBp50 is in both nucleus and cytoplasm (Figure 1a, lower panel), while PPARγ is found accumulated in the cytoplasm. Fluorescence intensity was quantified in both cell compartments and represented in Figure 1b. Meanwhile, Figure 1c shows the quantification of cell nuclei positive for the double labeling PPARγ/NF-kBp50, and it is observed that both factors are mostly expressed in the nucleus of normal liver tissue.

### 3.2. Pirfenidone Prevents Weight Loss Induced by Hepatotoxic Damage and Upholds Healthy Liver Morphology

**Figure 2:**
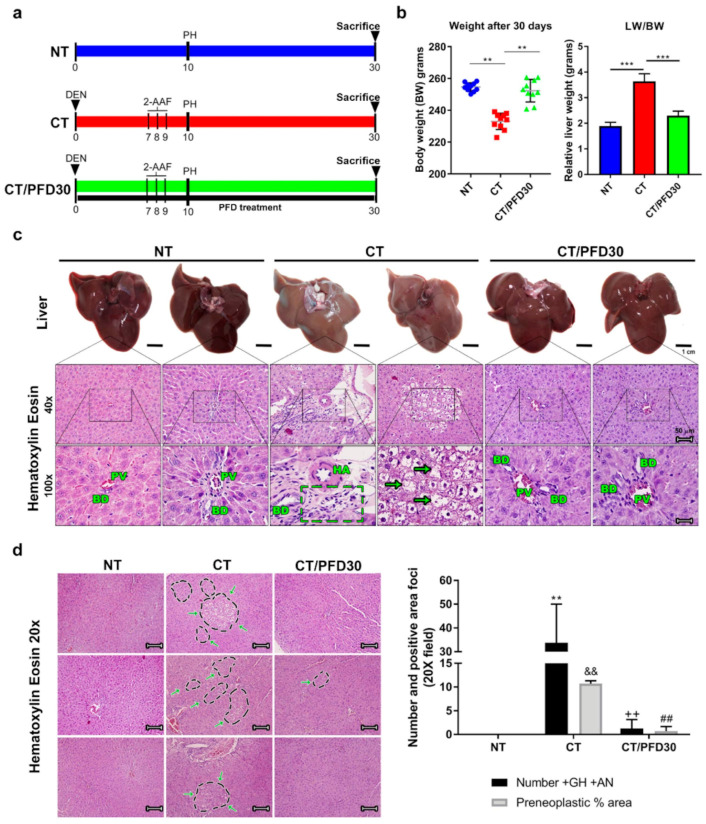
PFD administration can prevent chemical-induced pathological damage. (**a**) Experimental design. NT: No-treatment group; CT: Complete carcinogenic treatment group (MRHM protocol); CT/PFD30: Complete carcinogenic treatment plus PFD concomitant administration for 30 days. (**b**) Weight of animals after 30 days under experimental MRHM protocol. (**c**) Representative liver images in experimental groups after 30 days and H&E stain panel. (**d**) Representative graph of the number of positive atypical nuclei and preneoplastic lesions; +GH: positive giant hepatocytes; +AN: positive atypical nuclei. Scale bar 1 cm and H&E 50 µM. PV: portal vein; BD: bile duct; HA: hepatic artery. Inflammatory cell infiltrate (arrows). Preneoplastic lesions are evident (dotted rectangle). Arrow shows binuclear cell. Data are represented as mean ± SD. ** *p* < 0.01, *** *p* < 0.001; ** *p* < 0.01 vs. NT group; && *p* < 0.01 vs. NT group; ++ *p* < 0.01 vs. CT group; ## *p* < 0.01 vs. CT group.

Chemical-induced damage effect resulted in a significant weight decrease (Figure 2b), morphological liver alterations, and histological architecture in CT group (Figure 2c). Pale, swollen and dense livers are shown (Figure 2c), while H&E shows a significant inflammatory cell infiltrate, portal triad deformity (dotted rectangle), and binucleated cells (arrow). Hepatocytes were observed altered, basophilic, and translucent with prominent nucleus and nucleoli, a hallmark of preneoplastic lesions (number symbol) in CT group, while the liver macroscopic and histological analysis showed similarities between NT and CT/PFD30, both without preneoplastic lesions, depicting a maintenance of portal triads and normal sinusoids (Figure 2c). In agreement with this result, number of giant hepatocytes (+GH) and atypical nuclei (+AN) was determined in the H&E stains. Tissue sections of the CT group showed a high number of positive fields + GH and + AN (arrows, Figure 2c); on the contrary, PFD treatment was effective in preventing positive fields of +GH and +AN (Figure 2d, ++ *p* < 0.01 vs. CT; ## *p* < 0.01 vs. CT). Weight of rats of CT/PFD30 group was significantly different than those in CT group, PFD administration prevented weight loss in MRHM (Figure 2b); in addition, treatment of rats with PFD significantly attenuated MRHM-induced increases in relative liver weight Figure 2b and Table S4 in Supplementary Material. Lastly, biochemical markers only show significant differences of aspartate aminotransferase and alkaline phosphatase between CT/PFD30 and CT groups (Table 1 and Table S5 in Supplementary Material).

### 3.3. Fibrosis-Induction Is Prevented by PFD Administration in the MRHM

**Figure 3:**
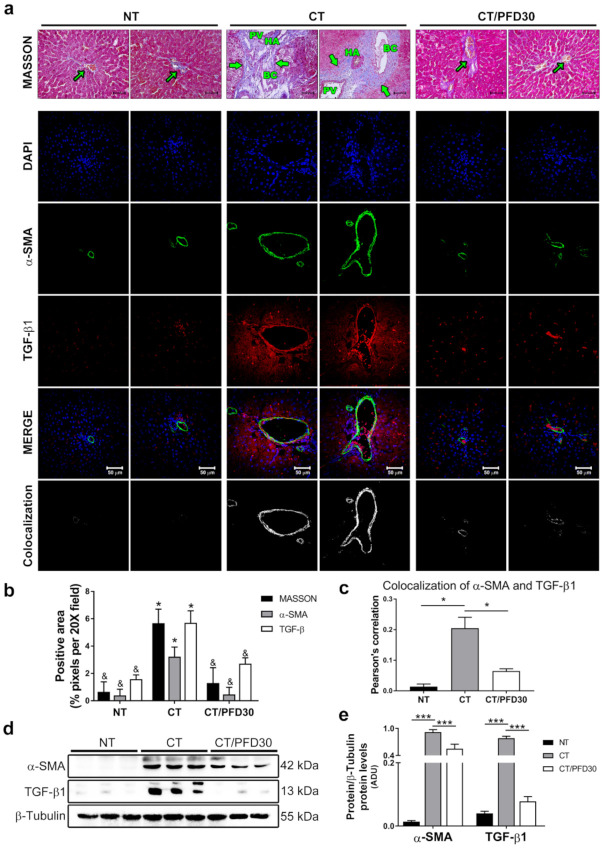
Liver fibrosis reduction by pirfenidone in MRHM. (**a**) Masson´s staining and IF for α-SMA (green), TGF-β1 (red), and DAPI (blue) in study groups. (**b**) Quantification of extracellular matrix by Masson´s and quantification of positive area of α-SMA/TGF-β by (**c**) Pearson’s correlation of co-localization for TGF-β and α-SMA. (**d**) Western-blot of α-SMA and TGF-β1 expression in liver tissue. (**e**) Graph of relative quantification of α-SMA and TGF-β1 expression in liver tissue. Data is represented as mean ± SD. NT: Non-treatment group. CT: Complete carcinogenic treatment group. CT/PFD30: Complete carcinogenic treatment plus PFD administration. PV: portal vein. BC: bile duct. HA: hepatic artery. * *p* < 0.05, *** *p* < 0.001, & no significant difference. Arrows show extracellular matrix.

Masson’s staining shows higher amount of extracellular matrix in MRHM (asterisk) rats, but fibrogenesis is prevented with concomitant PFD administration. Fibrosis values were quantified and significance for liver fibrosis were observed in CT vs. CT/PFD group (Figure 3a). NT and CT/PFD30 groups had similarities in histology (arrows), undistorted portal triad, and basal matrix positive staining, which demonstrated a preventive anti-fibrotic role of PFD in MRHM. To corroborate PFD preventive role IF assays and Western-Blots (WB) were performed. Figure 3a-IF panel showed α-SMA and TGF-β1 expression in rat liver; α-SMA and TGF-β1 overexpression were highly significant in CT group vs. NT (*p* < 0.05) and CT group vs. CT/PFD30 (Figure 3b,d,e), this last group showed a similar expression for NT group (CT/PFD30 vs. NT). In the same way, cytoplasmic protein expression of α-SMA and TGF-β1 were higher in CT group vs. NT (*p* < 0.001). However, rats on CT/PFD30 displayed lesser pro-fibrotic protein overexpression, supporting the anti-fibrotic effect of PDF (Figure 3c, *p* < 0.001). Finally, Pearson’s correlation for α-SMA and TGF-β1 co-localization is significant for CT group vs. CT/PFD30, where both proteins expression showed a positive correlation; NT group values show *p* > 0.05.

### 3.4. Inflammation Is Modulated by the Pirfenidone-Induced NF-kB p65/p50 Pathway

**Figure 4:**
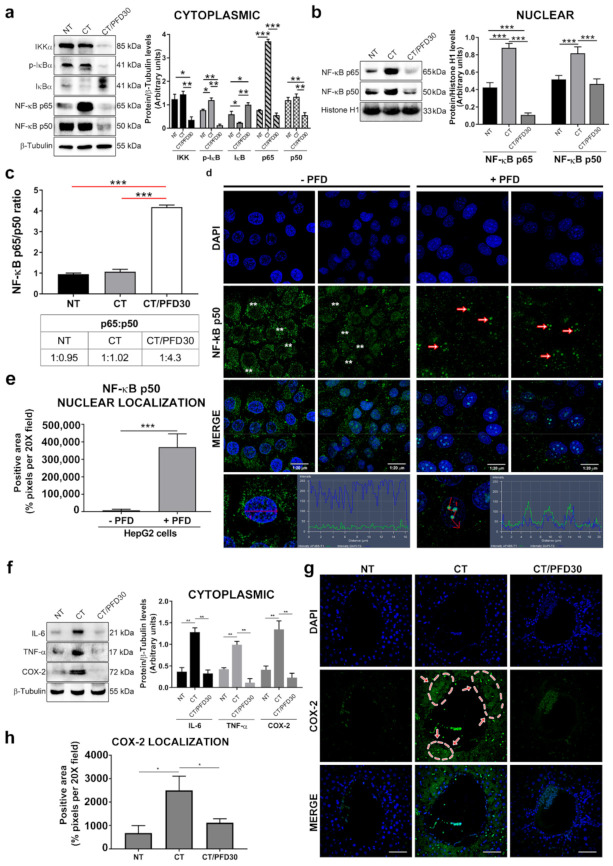
Pirfenidone reduced inflammation in MRHM and modified NF-kB p65/p50 ratio. (**a**) NF-kB activation cytoplasm cascade: IKK-α, p-IkB-α, IkB-α and NF-kB p65/p50 expression. (**b**) NF-kB p65/p50 nuclear expression. (**c**) NF-kB p65/p50 ratio values (**d**) Upper: IF for NF-kB p50 (green) and DAPI (blue) in HepG2 cells treated and non-treated with PFD. Asterisks indicate the cytoplasmic expression of NF-kB, arrows show NF-kB p50 nuclear clusters in cells treated with PFD. 1:20 µm scale bar. Lower panel: analysis of fluorescence of nuclear intensity for NF-kB p50; PFD-treated cells accumulate higher euchromatin clusters. (**e**) IF positive area values to NF-kb p50 (**f**) IL-6, TNFα and COX-2 cytoplasm expression. (**g**) IF for COX-2 (green) and DAPI (blue); microscopic fields depicting inflammation positive (arrows) analyzed by confocal microscopy; 50 µm scale bars. (**h**) Positive area values for COX-2. All bars represent the average value of SD of all rats in the group. NT: No-treatment group; CT: Complete carcinogenic treatment group; CT/PFD30: Complete carcinogenic treatment plus PFD administration; PFD: pirfenidone * *p* < 0.05. ** *p* < 0.01. *** *p* < 0.001.

Anti-inflammatory effects of PFD on liver damage and the expression and location of NF-kB p65/p50 heterodimers in cytoplasmic and nuclear fractions were analyzed. Figure 4a shows NF-kB p65/p50 activation cascade. CT group showed an overexpression of IKKα and increased IkB-α phosphorylation (p-IkBα) due to hepatocarcinogenesis damage. On the other hand, CT/PFD30 group experienced lower expression of both proteins, resulting in an increased IkB-α cytoplasmic accumulation, suggesting that NF-kB is kept in its inactive form (Figure 4a). Consequently, in this activation cascade, NF-kB heterodimer nuclear expression was elucidated in Figure 4b. CT group had a higher expression of both proteins. However, PFD was effective to prevent NF-kB p65 overexpression (CT/PFD30 vs. CT, *p* < 0.001), highlighting the restrictive PFD role in the translocation of NF-kB p65 to the nucleus and, therefore, preventing inflammation. Additionally, NF-kB p65/p50 ratio was analyzed (Figure 4c, red line); this quotient indicated a value of 1:4.3 (*p* < 0.01), which suggests the formation of NF-kB p50 homodimers by the nuclear prevalence of p50. To further corroborate this finding, HepG2 cells were subjected to treatment with and without PFD. Results showed NF-kB p50 overexpression and chromatin clusters in cells treated with PFD (Figure 4d), the positive area was significant (-PFD vs. +PFD *p* < 0.001 Figure 4e). Lastly, TNFα, IL-6, and COX-2 proteins were analyzed in the cytoplasm as targets of NF-kB transcriptional activity. Figure 4f shows TNFα, IL-6, and COX-2 expression in CT group, PDF treatment was effective to prevent the expression of these proteins (CT/PFD30 vs. CT, *p* < 0.01). Additionally, Figure 4g,h shows IF, intensity quantification and positive area forCOX-2. NT group displayed lesser location of COX-2 compared to CT group which had overexpression of this protein. PFD treated rats was effective to prevent COX-2 expression (CT vs. CT/PFD30, *p* < 0.05).

### 3.5. Increased Expression of Apoptotic Markers in Nuclear and Cytoplasmic Fractions by Pirfenidone

**Figure 5:**
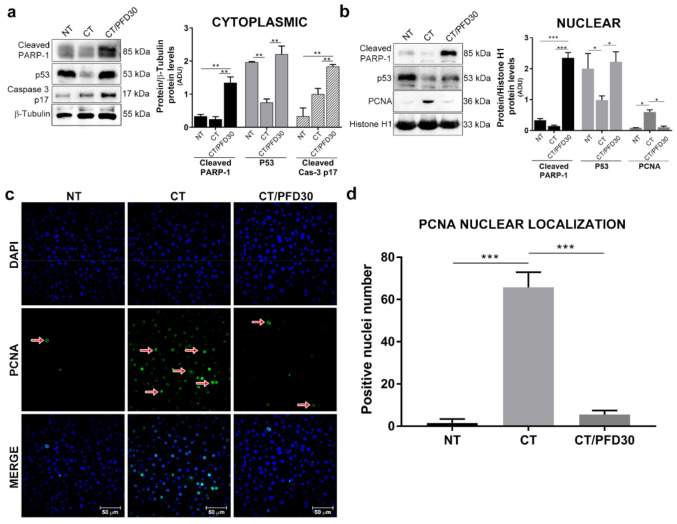
Activation markers of pro-apoptotic pathway in MRHM by PFD. (**a**) PARP-1 p85, p53 and caspase-3 p17 expression in cytoplasm. (**b**) PARP-1 p85, p53 and PCNA nuclear expression. (**c**) IF for PCNA (green) and DAPI (blue). 50 µm scale. (**d**) Fluorescence intensity and quantification of positive area in histological sections for PCNA. Arrows show cells with intensity for PCNA. Signal intensities were determined by densitometric analysis and values calculated as the ratio of protein of interest to histone H1 or β-tubulin. Each bar represents the average value of ten rats. NT: No-treatment group; CT: Complete carcinogenic treatment group; CT/PFD30: Complete carcinogenic treatment plus PFD administration. * *p* < 0.05. ** *p* < 0.01. *** *p* < 0.001.

Apoptosis evasion plays an important role in cells initiated with carcinogenic damage. Figure 5a,b shows a p53 overexpression in the cytoplasm and nucleus in NT and CT/PFD30 groups compared to CT group (CT vs. NT and CT/PFD30 *p* < 0.01). In the same way, Caspase-3 p17 (active form) in the cytoplasm was evaluated; data shows that only CT/PFD30 group has a significant caspase-3 p17 accumulation in the cytoplasm compared to the other groups (*p* < 0.01). To corroborate this effect, truncated PARP-1 was analyzed, whose85 kDa fragment corresponds to the nonfunctional form of DNA repair and is related to the active pathway of apoptosis; the results showed a significant truncated PARP-1 in both cytoplasm and nucleus of CT/PFD30 group, (CT/PFD30 vs. CT and NT ** *p* < 0.01, *** *p* < 0.001). The PCNA proliferation marker was found increased in CT group in nuclear extracts as shown in Figure 5b; though PFD prevented the occurrence of this marker. To confirm this result, a confocal microscopy analysis was carried out (Figure 5c), where the positive area of PCNA expression in CT group (arrows), was compared with NT and CT/PFD30 groups (*p* < 0.001). PFD was effective in preventing PCNA expression (Figure 5d) and stimulating apoptosis response in MRHM.

### 3.6. Pirfenidone Promotes PPARγ Expression and Its Translocation to Nuclei

**Figure 6:**
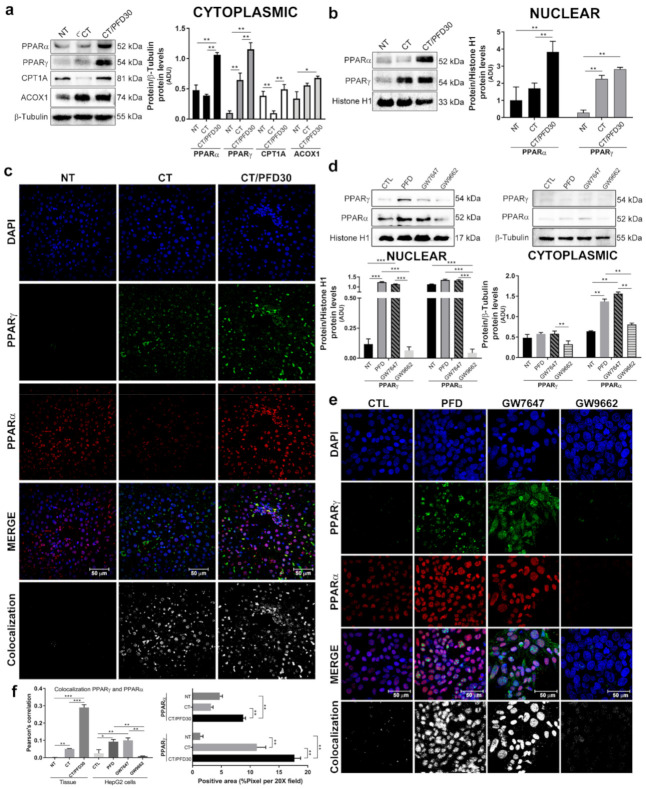
PFD administration induces overexpression and nuclear translocation of PPARα and PPARγ in the MRHM and HepG2 cells. (**a**) PPARα, PPARγ, CPT-1 and ACOX-1 cytoplasm expression. (**b**) PPARα and PPARγ nuclear expression. (**c**) IF for PPARα (red), PPARγ (green) and DAPI (blue) in tissues from MRHM. (**d**) PPARα and PPARγ in nucleus and cytoplasm expression respectively in HepG2 cells treated with PFD 500 µM, GW7647 1 μM and GW9662 100 nM. (**e**) IF for PPARα (red), PPARγ (green) and DAPI (blue) in HepG2 cells treated with PFD, GW7647 and GW9662. (**f**) Left panel: Pearson’s correlation of co-localization for PPARγ/PPARα in rats from MRHM and HepG2 cells treated with PFD, GW7647 and GW9662. Right panel: Quantification of positive area in histological sections for PPARα and PPARγ; 50 µm scale bars. Each bar represents the average value of ten rats or tripled HepG2 cells treatment. NT: No-treatment group; CT: Complete carcinogenic treatment group; CT/PFD30: Complete carcinogenic treatment plus PFD administration; PFD: pirfenidone. * *p* < 0.05. ** *p* < 0.01. *** *p* < 0.001.

Figure 6 shows the expression, translocation, and co-localization panels of PPARα and PPARγ proteins. In the cytoplasm, PPARα and PPARγ expression in CT/PFD30 group was significantly higher compared to the NT and CT groups (*p* < 0.01). The effect of PFD administration on hepatic lipid β-oxidation was evaluated by PPARα signaling pathway key proteins such as CPT1A and ACOX1. CPT1A expression decreased in the CT group, while in CT/PFD30 group the expression of this protein was maintained at normal steady levels (*p* < 0.01). ACOX1 resulted overexpressed under the effect of PFD (*p* < 0.05). In the nucleus, PPARα expression was significantly higher in CT/PFD30 group compared to NT and CT groups (Figure 6b, *p* < 0.01). IF panel indicates a greater number PPARα-positive fields in the nucleus from CT/PFD30 group (shown in Figure 6c, f right graph, *p* < 0.01). Meanwhile, PPARγ prominent nuclear expression in both CT and CT/PFD30 groups determined by WB correlated with IF analysis showing positive fields vs. NT group (Figure 6c,f right graph *p* < 0.01). To further corroborate and extend our findings on the effect of PFD on PPARs, in vitro experiments were carried out using HepG2 cells treated with PPARγ agonists and antagonists, respectively. In Figure 6d,e, PFD treatment induces an increase in both nuclear PPARs-expression, while in cytoplasmic this effect was not observed. Besides, it can be seen in Figure 6e,f that PFD treatment induces a comparable translocation of PPARγ as the agonist GW7647. Contrariwise, cells treated with PPARγ antagonist GW9662 inhibited the expression and nuclear translocation of both PPARs.

### 3.7. In-Silico Assay Demonstrate That Pirfenidone Is a PPARγ Ligand

**Figure 7:**
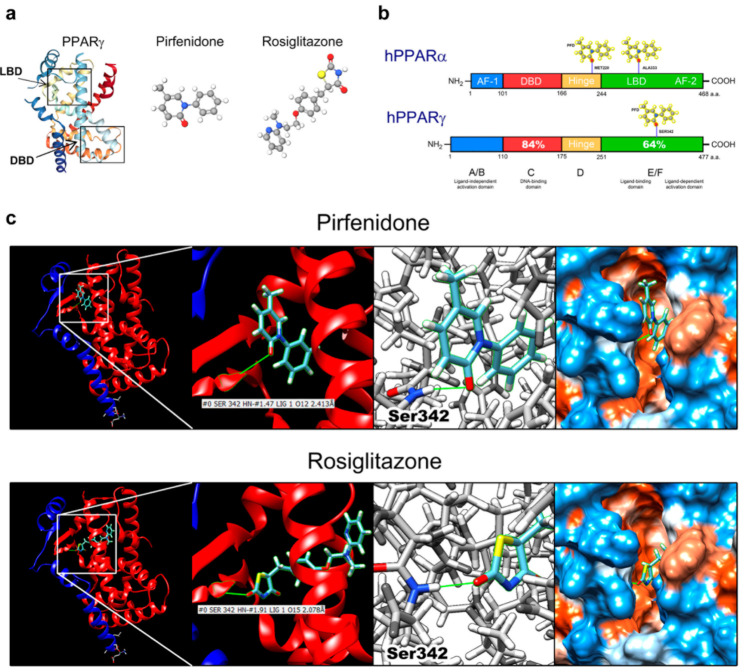
Pirfenidone is a ligand/agonist for PPARγ. (**a**) Representative images of PPARγ with LBD obtained from PDB. (**b**) Linear image of PPARα and PPARγ showing the homology between these proteins and probable PFD binding sites. Percentage of 64% homology in LBD between proteins is shown. (**c**) In silico assay with PFD and rosiglitazone with PPARγ LBD. LBD: ligand-binding domain; DBD: DNA-binding domain.

Molecular docking assay for binding between PFD and PPARγ at its ligand-binding domain was performed. LBD and DNA-binding domain regions of PPARγ, as well as PFD and rosiglitazone are represented in Figure 7a. As previously described, PPARγ and PPARα share high homology, displaying 64% homology in their LBD (Figure 7b). Here, in silico assays demonstrated that the oxygen-12 of PFD forms a hydrogen bond (Å: 2.413) with the nitrogen of Ser342 in PPARγ-LBD, with a ΔG: −6.99 (Figure 7b). Comparing with rosiglitazone as a selective bona fide PPARγ agonist, it was shown that PFD binds to the same Ser342 residue (Figure 7b). To demonstrate this specific binding, an in-silico mutagenesis of serine for glycine in the native sequence of PPARγ was performed, wherein PFD-PPARγ binding is broken up at the Gly342. Therefore, it is site-specific for serine 342 (Supplementary Figure S1).

## 4. Discussion

In human HCC, studies concerning PPARγ expression are controversial. However, Schaefer et al., demonstrated that PPARγ is overexpressed in neoplastic lesions [24]. Recently, Afaloniati et al., confirmed in a HCC murine model, that treatment with romidepsin, an HDACs inhibitor, can modulate the translocation of PPARγ to the nucleus, proposing this transcriptional factor as a possible anti-inflammatory mediator [25]. Our analysis in normal human liver showed that there is a basal nuclear expression of PPARγ, while in human HCC tissue exists and cytoplasmic PPARγ overexpression. This response could be associated with an increase in cell proliferation and nullified apoptosis in human HCC [24].

Experimental liver damage models are useful tools for the study of the pathophysiology of several diseases including HCC [12]. MRHM allows to study initiation-promotion stages of this disease, which were observed in this study and prevented by PFD administration.

Liver fibrosis plays an important role in HCC-development, TGF-β modulates processes such as cell invasion, and cellular microenvironment modification that cancer cells could utilize to their benefit [26]. The TGFβ pathway has pleiotropic functions regulating cell growth, differentiation, apoptosis, motility and invasion, extracellular matrix production, angiogenesis, and immune response. Deregulation of the TGFβ pathway is common in tumors and plays a critical role in tumor initiation, development, and metastasis, and accumulation of genetic alterations in the TGFβ pathway drives pathway evolution from tumor suppressive to tumor promoting activities [26]. Our data provide evidence that PFD prevents histopathological alterations caused by collagen accumulation through inhibiting TGF-β1 and α-SMA overexpression, avoiding extracellular matrix accumulation. Anti-fibrotic activity of PFD is well documented and demonstrated from results obtained in animal models and clinical trials of liver damage, however, in these previous publication TGF-β1 expression occurred up to 8 weeks after the induced damage, and no carcinogenic lesions development was reported in these studies. [15,16]. MRHM induces several changes at the genomic and tissue level, from fibrosis to preneoplastic injury, facilitating the mesenchymal/epithelial transition from the beginning of liver damage up to HCC progression [27]. TGF-β1 plays an important role in controlling the growth and death of hepatocytes and liver tumor cells [28,29]. TGF-β overexpression activates proliferative and antiapoptotic signals through transactivation of platelet derived growth factor (PDGF) or epidermal growth factor (EGF) signaling [30]. For all those reasons, TGF-β1 inhibition might result in an early mechanism to prevent the development of initial HCC as observed in this study.

On the other hand, the inflammatory response is an active process that participates in carcinogenic damage progression [2]. NF-kB pathway has long been considered a typical proinflammatory mechanism, with the ability to promote proinflammatory gene expression including cytokines, chemokines, and adhesion molecules [31]. Several drugs such as S-adenosylmethionine, N-acetylcysteine, and quercetin have demonstrated a chemoprotective effect through NF-kB modulation and their antioxidant abilities [32]. In this study, PFD administration was able to prevent NF-kB expression, and their nuclear translocation, besides that modifying p65/p50 ratio proportion. Studies have indicated that NF-kB p65 is a key mediator in early events that promote neoplastic lesion progression in the liver. An aberrant overexpression of p65 and its subsequent signaling has been observed in both human HCC tissue and cell lines (HepG2 and HepG3) [31]. Additionally, IkB-α overexpression has been shown to inhibit p65 modulated signaling [33]. Drugs such as sorafenib, a multikinase inhibitor used for HCC-treatment, decrease p65 expression and its activity, resulting in downregulation of molecular intermediaries for the promotion, proliferation, and expression of cell invasion [34]. Our results demonstrated that PFD stimulates the expression of IkB-α, which in turn inhibits p65 nuclear translocation, preventing an upregulation of IL-6, TNFα, and COX-2 (Figure 4f). Additionally, IkB-α promoter is regulated by PPARα and PPARγ expression [35], which leads to increased IkB-α transcription and, therefore, IkB-α-p65 binding. Our experiments suggest that PFD induces PPARγ nuclear translocation, which in turn could favor overexpression of IkB-α, and inhibition of p65 and their target genes. In addition, PFD decreased IkB-α phosphorylation, and therefore minimizes p65-released available to migrate to the nucleus.

On the other hand, Yu et al., analyzed the biological function of p50 subunit, proposing it as a potential therapeutic target in the treatment of cancer. NF-kB isoform p105 is truncated by 26S proteasome action, generating a p50 subunit, which lacks intrinsic transcriptional activity; this transcriptional activity is acquired by forming heterodimers with p65, Rel B or C-Rel subunits, to subsequently translocate to the nucleus [36]. The p65/p50 heterodimer is considered a potent activator of pro-tumoral genes such as: IL-6, cyclin-D, VEGF, and MMP-9 [37]. Conversely, the p50 homodimer acts in the nucleus as a repressor of the inflammatory response, and consequently might circumvent cancer [36,37]. Our work confirms that in the damage generated exists an increase in p65/p50 expression, in both, cytoplasm and nucleus (Figure 4a,b). Interestingly, PFD induces p50 nuclear translocation, modifying p65/p50 ratio in favor of p50, preventing IL-6, TNFα, and COX-2 expression.

For its part, COX-2 is a proinflammatory enzyme involved in cell proliferation, tumorigenesis, progression, and metastasis. It has been observed that an increase of levels of this protein may constitute a mechanism that facilitates carcinogenesis; therefore, the prevention of this response might have positive effects against HCC-progression [38]. In this work, the results showed that PFD was able to prevent COX-2 expression and localization in bile.

Regarding apoptosis this is a process orchestrated by a series of stimuli, which include p53-activation and proteolysis of pro-caspase 3 to caspase 3-p17 [39,40]. Previous reports indicate that PPARγ ligands increase its recruitment to the p53 promoter sequence, rising its transcription [41,42]. Furthermore, PPARγ and p53 are related to triggering apoptosis by cleavage of caspase-9 and DNA fragmentation [43]. Recently Cheung KF et al., demonstrated that PPARγ overexpression in hepatoma cells showed significant inhibition of cell growth, increased cell apoptosis through intrinsic (caspase-3-p17, 7, 9 and PARP) and extrinsic (Fas, TNF-α, and caspase-8) pathways [43]. The evidence establishes a functional link between PPARγ and p53-dependent signaling. Our data showed that PFD clearly increased p53 and caspase 3-p17.

The role of PCNA is also important since this is a protein involved in synthesis and repair of DNA [44], Venturi et al. showed that PCNA immunostaining in cancerous and surrounding cirrhotic livers was selectively localized in the nucleus and mainly in hepatocytes, with few cases showing weak positivity in fibroblasts and biliocytes [44], very similar to our immunofluorescence findings, in where the PCNA localization was present in the nucleus of liver tissues of CT group, but this response was no affected by PFD treatment. In addition, Mun et al. made the determination of immunopositive cells in HCC livers, where they found that PCNA expression in HCC liver is increased in up to 73% of cells per field [45], data like what we found, where the number of positive cells for PCNA it high. Shu et al., found that PPARγ phosphorylation has effects on PCNA active levels, PPARγ expression was inversely proportional to PCNA, suggesting a possible mechanism for controlling proliferation by PPARγ [46]. Data coincides with our findings where PFD was effective to activate PPARγ and preventing PCNA overexpression (Figure 5 and Figure 6).

Finally, structural studies and dynamic mechanism of PPARγ showed that Ser342 in LBD is the most important amino acid for the binding of other partial agonists such as GW0072 and GQ-16.47 We demonstrated that PFD could form a hydrogen bond with Ser342 with a similar Gibbs-free energy as rosiglitazone, an agonist for PPARγ [47]. Our data clearly showed that PFD exhibited early antitumor activity in addition to anti-fibrotic and anti-inflammatory effects [15,16].

## 5. Conclusions

**Figure 8:**
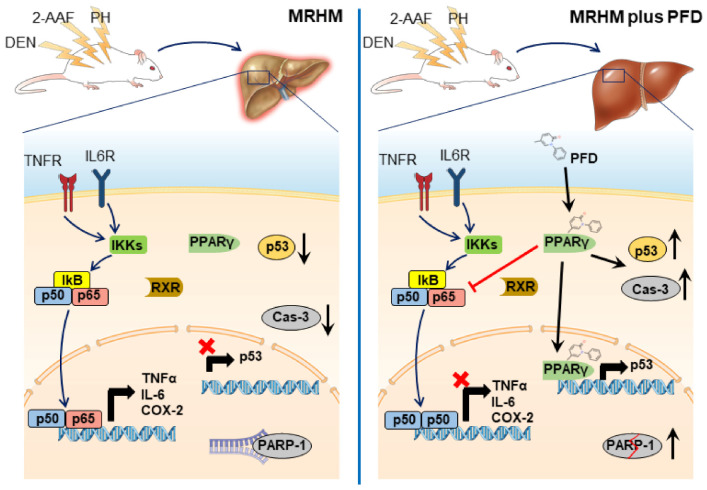
Summary mechanism of action of PFD in the early stages of HCC. Left panel: Molecular mechanisms activated in the development of HCC; transduction of proinflammatory genes regulated by the NF-kB p65/p50 heterodimer. Right panel: PFD is a ligand/agonist of PPARγ activating it signaling pathway and modifying NF-kB p65/p50 translocation, preventing inflammation, and increasing p53 activity and caspase 3 activation, avoiding HCC development.

Our data demonstrate for the first time PFD effects on key proteins in fibrosis, inflammation, and cell proliferation in an experimental model that replicates the initial stages of HCC. The possible mechanism of action of PFD in MMHR is summarized in Figure 8. However, additional studies are necessary that can assess the long-term anti-tumor effects.

## Figures and Tables

**Table 1:** Effect of PDF (30 days at 500 mg/kg) on serum markers of liver damage.

Biochemical Marker	NT	CT	CT/PFD30	*p*
AST (U/L)	162.50 ± 11.42	123.50 ± 16.26	166.50 ± 15.76	<0.01
ALT (U/dL)	74.50 ± 6.36	82.00 ± 1.41	81.00 ± 5.66	NS
Alkaline Phosphatase (U/L)	338 ± 9.90	442 ± 25.46	301 ± 65.05	<0.05

Values are expressed as means ± SD (*n* = 10). AST: aspartate aminotransferase; ALT: alanine aminotransferase.

## Data Availability

All data generated and analyzed during the present study are included in this published article.

## Supplementary Materials

The following are available online at https://www.mdpi.com/article/10.3390/ijms222111360/s1.; Figure S1: Pirfenidone misses binding affinity to PPARγ mutated in SER342 by GLY342; Table S1: Histopathological characteristics of donated human HCC tissue. Table S2: Reagents used for cytoplasmic and nuclear fractions; Table S3: Antibodies list employed in the different methodologies; Table S4: Effect of PFD (30 days at 500 mg/kg), on relative liver weight; Table S5: Effect of PDF (30 days at 500 mg/kg) on serum markers of liver damage (complete)and entire blots from Western Blots presented in the figures.
