# Supplementary material for: Hepatocarcinogenesis Prevention by Pirfenidone Is PPARγ Mediated and Involves Modification of Nuclear NF-kB p65/p50 Ratio
Source: Int J Mol Sci. 2021 Oct 21;22(21):11360. doi: 10.3390/ijms222111360 (PMC8583060; doi:10.3390/ijms222111360)

Hepatocarcinogenesis prevention by pirfenidone is PPARγ mediated and involves modification of nuclear NF-kB p65/p50 ratio.

Jorge A. Silva-Gomez, Marina Galicia-Moreno, Ana Sandoval-Rodriguez, Arturo Santos, Hugo C. Monroy-Ramírez and Juan Armendariz-Borunda.

1. Supplementary material and methods.

1.1. Animals.

Male Fisher rats weighing 180g, were distributed in three groups (shown in Fig. 1a): Non-treated group (**NT**) (n=10), complete treatment for induction of HCC group (**CT**) (n=10) and complete treatment plus PFD administration for thirty days (**CT/PFD30**) (n=10). CT and CT/PFD30 groups were subject to MRHM, and NT group was used as a control. Rats were provided by UPAE-Bioterio at CUCS, University of Guadalajara, Mexico, carried out in accordance with institutional guidelines under bioethical and research approval number CI-03020, and additional, conformed to ARRIVE guidelines. All animals were maintained at 25±2°C with 12-hour light/dark cycles with food and water *ad libitum*.

1.2. Hepatectomy for MRHM design.

All rats were subjected to 4-hour prior fast to perform surgery. Procedure consisted first in anesthesic administration with Zoletil 100 mg (tyletamine with zolazepam) at 15 IU dose for every 200 grams of weight of each rat by intraperitoneal administration (30-50mg/kg). Complete disinfection of anterior side of each animal using 80% benzalkonium chloride was performed; later, an incision with a 21 scalpel of approximately 2 cm was made in ventral plane with a right flank tendency was made, and the hepatic lobes were shown by abdominal pressure. A 4-0 chromic catgut ligation was performed for the right liver log and 30% of the left lobe. The rest of the left lobe and caudate process were not removed. Finally, the muscular and epidermal layers of each animal were sutured and kept under observation for 8 hours [1,2].

1.3. Biochemical measurements.

On day 30 during the sacrifice and under anesthesia, a cardiac puncture was performed to obtain blood. The blood samples were taken and immediately centrifuged at 3500 rpm for 15 minutes to obtain serum. Biochemical variables were performed using adry chemistry analyzer (Vitros 250 Analyzer, Ortho-Clinical Diagnostics, Johnson & Johnson Services, Inc.,Rochester, NY, USA).

1.4. Protein extraction and Western Blot.

For protein extraction, 100mg of liver tissue from each of the animals in the 3 groups and triplicated treated cell samples were used. Samples were homogenized with pestle homogenizer using a 1 ml of cytoplasm extraction buffer for tissue and 100µl for cells (Supplementary Table 2) with a protease inhibitor cocktail to isolate the cytoplasm fraction at 17,000 rpm at 4°C for 15 minutes. Then, nucleus extraction buffer (Supplementary Table 1) with protease inhibitor cocktail was

incorporated to pellet, vortex 30 min in a cold room and centrifuged at 17,000 rpm at 4°C for 15 min to obtain the nucleus fraction. The supernatant was recovered in both fractions and total protein quantified by mini-Bradford [3]. Proteins were boiled 5 min in Laemmli Sample Buffer 2X. 50μg of each sample were separated by SDS-PAGE in acrylamide gels. Proteins were electro-transferred to PVDF membranes. Membranes were then incubated at 4°C overnight, with the corresponding primary antibody (Supplementary table 2) and with secondary antibody (Anti-Mouse/anti-Rabbit IgG-POD, BM Chemiluminescence Western Blotting Kit Mouse/Rabbit, Roche). Bands of interest were visualized using BioRadChemiDoc™ XRS+ System software.

1.5. Immunofluorescence.

Liver tissues were collected on day 30 and sliced into 4μm-thick sections, deparaffinized and permeabilized in PBS with 0.2%TritonX100 and treated for 1 min with 0.1% Sudan Black B (Santa Cruz Biotechnology, Santa Cruz, CA) to reduce tissue auto-fluorescence. Subsequently, all sections were blocked with 5% BSA and incubated with primary antibody (supplementary table 2) and goat anti-rabbit IgG Alexa Fluor-488 (1:200) and Alexa Fluor-594 (1:200) (Jackson Immunoresearch, West Grove, PA). Nucleus were stained with DAPI (4′,6-diamidino-2-phenylindole) (Molecular Probes, Eugene, OR, USA) (1:5000). As a control for antibody specificity, all liver tissues were incubated with a rabbit primary antibody isotype control (Invitrogen). Liver tissues and cells were analyzed by confocal microscopy using a ZEISS laser-scanning microscope LSM 800 at EC Plan-Neofluar 20x/0.50 M27, Plan-APO 40x/1.3 Oil DIC III and Plan-Apochromat 63x/1.40 Oil DICM27 objectives. Maximum projection and intensity of fluorescence was analyzed with free access software ZEN 2.3 SP1.

2. References.

1. Semple-Roberts E, Hayes MA, Armstrong D, Becker RA, Racz WJ, Farber E. Alternative methods of selecting rat hepatocellular nodules resistant to 2-acetylaminofluorene. Int J Cancer. 1987; 40: 643-5.
2. Carrasco-Torres G, Monroy-Ramírez HC, Martínez-Guerra AA, Baltiérrez-Hoyos R, Romero-Tlalolini MLÁ, Villa-Treviño S, et al. Quercetin Reverses Rat Liver Preneoplastic Lesions Induced by Chemical Carcinogenesis. Oxid Med Cell Longev. 2017; 2017: 4674918.
3. Bradford MM. A rapid and sensitive method for the quantitation of microgram quantities of protein utilizing the principle of protein-dye binding. Anal Biochem. 1976; 72: 248-54.

3. Supplementary tables.

Supplementary Table S1: Histopathological characteristics of donated human hcc tissue.

Code of tissue	SEX	AGE	DIAGNOSTIC
Qx-4870-18*	Masc	57	Moderately differentiated hepatocarcinoma
Qx-3357-19	Masc	59	Poorly differentiated hepatocarcinoma
Qx-4832-19*	Masc	29	Moderately differentiated hepatocarcinoma
Qx-5131-19	Fem	65	Well-differentiated hepatocarcinoma
Qx-1898-20	Masc	42	Moderately differentiated hepatocarcinoma
Qx-2022-20	Fem	60	Moderately differentiated hepatocarcinoma
Qx-2589-20	Masc	53	Poorly differentiated hepatocarcinoma
Qx-2965-20*	Masc	67	Moderately differentiated hepatocarcinoma
Qx-3721-20*	Fem	66	Moderately differentiated hepatocarcinoma
Qx-3780-20*	Masc	20	Moderately differentiated hepatocarcinoma
Qx-4431-20*	Masc	62	Moderately differentiated hepatocarcinoma

*** Main tissues analyzed.**

Supplementary Table S2: Reagents used for cytoplasmic and nuclear fractions.

**Reactive**	**Concentration for cytoplasmic fraction**	**Concentration for nuclear fraction**
HEPES	10 mM	20 mM
KCl	10 mM	---
NaCl	---	400 mM
EDTA	0.1 mM	0.1 mM
NP-40	0.5%	---
DTT	1 mM	1 mM
NAF	0.05 M	0.05 M
Na3VO4	0.2 mM	0.2 mM
PMSF	1 mM	1 mM

HEPES: (4-(2-hydroxyethyl)-1-piperazineethanesulfonic acid); KCl: potassium chloride; NaCl: sodium chloride; EDTA: Ethylenediaminetetraacetic acid; NP-40: Nonidet-P40; DTT: Dithiothreitol; NAF: sodium fluoride; Na3VO4: sodium orthovanadate; PMSF: phenylmethylsulfonyl fluoride.

Supplementary Table S3. Antibodies list employed in the different methodologies.

Antibody	Epitope	Host-class	Procedure	Citation	Reference Provider
α-SMA	α-SMA	Rabbit IgG	WB IF	[1]	Cell Signaling, Danvers, MA
ACOX-1	ACOX-1 361-649	Rabbit IgG	WB	[2]	Abcam, Cambridge, MA
β-Tubulin	β-Tubulin 210-244	Mouse IgG	WB	[3]	Santa Cruz Biotechnology
Caspase-3 p17	Caspase-3 p17 56-104	Mouse IgG	WB IF	[4]	Santa Cruz Biotechnology
COX-2	COX-2 17-55	Mouse IgM	WB IF	[5]	Santa Cruz Biotechnology
CPT-1A	CPT-1A 621-634	Mouse IgG	WB	[6]	Abcam
Histone-H1	Histone-H1 22-47	Mouse IgG	WB	[7]	Santa Cruz Biotechnology
Histone-H3	Histone H3	Rabbit IgG	WB	[8]	GenTex
IkB-α	IkBα	Mouse IgG_{2b}	WB	[9]	Santa Cruz Biotechnology
p-IkB-α	phosphorylated Ser 32 IκB-α	Mouse IgG_{2b}	WB	[10]	Santa Cruz Biotechnology
IKK-α	amino acids 1-745 full length IKKα	Mouse IgG_{2b}	WB	[11]	Santa Cruz Biotechnology
IL-6	IL-6	Mouse IgG_{2b}	WB	[12]	Santa Cruz Biotechnology
NF-kB p50	NFkB p50 120-239	Mouse IgG	WB IF	[13]	Santa Cruz Biotechnology
NF-kB p65		Mouse IgG	WB	[14]	Cell Signaling
P53	P53 353-391	Mouse IgG	WB	[15]	Santa Cruz Biotechnology
PARP-1	PARP-1 764-1014	Mouse IgG_{2a}	WB	[16]	Santa Cruz Biotechnology
PCNA	PCNA	Mouse IgG_{2a}	WB IF	[17]	Santa Cruz Biotechnology
PPARα	PPAR-alpha 1-18	Rabbit IgG	WB IF	[18]	Abcam
PPARγ	PPARγ Ser 112	Rabbit IgG	WB IF	[19]	Cell Signaling
TGF-β1	TGF-β1	Mouse IgG	WB IF	[20]	Santa Cruz Biotechnology
TNFα	TNFα	Mouse IgG	WB	[21]	Santa Cruz Biotechnology

WB, Western blotting; IF, Immunofluorescence.

Supplementary Table S4. Effect of PFD (30 days at 500 mg/kg), on relative liver weight.

**Groups**	**Liver weight (g)**	**Body weight (BW) (g)**	**Relative liver weight (g/g BW)**
NT	4.83 ±0.34	254.61 ± 2.70	1.89 ±0.14
CT	8.18 ±0.47	233.04 ± 5.16	3.51 ±0.20****
CT/PFD30	5.99 ±0.51	252.32 ± 7.05	2.30 ±0.17***, ++++

Values are expressed as means ± SD (n=10), ***p<0.0001 *vs* NT; ***p<0.001 *vs* CT; ++++p<0.0001 *vs* NT.

Supplementary Table S5. Effect of PDF (30 days at 500 mg / kg) on serum markers of liver damage (complete)

**Biochemical marker**	**NT**	**CT**	**CT/PFD30**	***P***
Total Bilirubin (mg/dL)	0.08 ± 0.05	0.06 ± 0.02	0.07 ± 0.04	NS
Direct bilirubin (mg/dL)	0.05 ± 0.05	0.03 ± 0.01	0.04 ± 0.03	NS
Indirect bilirubin (mg/dL)	0.30 ± 0.01	0.30 ± 0.01	0.30 ± 0.01	NS
GGT (U/dL)	1.60 ± 0.85	1.50 ± 0.71	2.00 ± 1.41	NS
Total proteins (g/dL)	6.20 ± 0.28	6.35 ± 0.07	6.60 ± 0.42	NS
Albumin (g/dL)	2.95 ± 0.21	2.80 ± 0.01	3.00 ± 0.14	NS
Globulin (g/dL)	3.25 ± 0.07	3.55 ± 0.07	3.60 ± 0.28	NS
A/G	0.91 ± 0.04	0.79 ± 0.01	0.84 ± 0.02	NS

Values are expressed as means ± SD (n=10). AST: aspartate aminotransferase; ALT: alanine aminotransferase; GGT: gamma-glutamyl transferase.

References

1. Wang K, Yang X, Wu Z, Wang H, Li Q, Mei H, You R, Zhang Y. Dendrobium officinale Polysaccharide Protected CCl4-Induced Liver Fibrosis Through Intestinal Homeostasis and the LPS-TLR4-NF-κB Signaling Pathway. Front Pharmacol. 2020 Mar 12;11:240. doi: 10.3389/fphar.2020.00240. PMID: 32226380; PMCID: PMC7080991.
2. Goto T, Hirata M, Aoki Y, Iwase M, Takahashi H, Kim M, Li Y, Jheng HF, Nomura W, Takahashi N, Kim CS, Yu R, Seno S, Matsuda H, Aizawa-Abe M, Ebihara K, Itoh N, Kawada T. The hepatokine FGF21 is crucial for peroxisome proliferator-activated receptor-α agonist-induced amelioration of metabolic disorders in obese mice. J Biol Chem. 2017 Jun 2;292(22):9175-9190. doi: 10.1074/jbc.M116.767590. Epub 2017 Apr 12. PMID: 28404815; PMCID: PMC5454100.
3. Njah K, Chakraborty S, Qiu B, Arumugam S, Raju A, Pobbati AV, Lakshmanan M, Tergaonkar V, Thibault G, Wang X, Hong W. A Role of Agrin in Maintaining the Stability of Vascular Endothelial Growth Factor Receptor-2 during Tumor Angiogenesis. Cell Rep. 2019 Jul 23;28(4):949-965.e7. doi: 10.1016/j.celrep.2019.06.036. PMID: 31340156.
4. Silva-Hirschberg C, Hartman H, Stack S, Swenson S, Minea RO, Davitz MA, Chen TC, Schönthal AH. Cytotoxic impact of a perillyl alcohol-temozolomide conjugate, NEO212, on cutaneous T-cell lymphoma in vitro. Ther Adv Med Oncol. 2019 Dec 6;11:1758835919891567. doi: 10.1177/1758835919891567. PMID: 31839810; PMCID: PMC6900611.
5. Mattos RM, Machado DE, Perini JA, Alessandra-Perini J, Meireles da Costa NO, Wiecikowski AFDRO, Cabral KMDS, Takiya CM, Carvalho RS, Nasciutti LE. Galectin-3 plays an important role in endometriosis development and is a target to endometriosis treatment. Mol Cell Endocrinol. 2019 Apr 15;486:1-10. doi: 10.1016/j.mce.2019.02.007. Epub 2019 Feb 10. PMID: 30753853.
6. Busquets S, Pérez-Peiró M, Salazar-Degracia A, Argilés JM, Serpe R, Rojano-Toimil A, López-Soriano FJ, Barreiro E. Differential structural features in soleus and gastrocnemius of carnitine-treated cancer cachectic rats. J Cell Physiol. 2020 Jan;235(1):526-537. doi: 10.1002/jcp.28992. Epub 2019 Jun 26. PMID: 31241186.
7. Cammarota F, Conte A, Aversano A, Muto P, Ametrano G, Riccio P, Turano M, Valente V, Delrio P, Izzo P, Pierantoni GM, De Rosa M. Lithium chloride increases sensitivity to photon irradiation treatment in primary mesenchymal colon cancer cells. Mol Med Rep. 2020 Mar;21(3):1501-1508. doi: 10.3892/mmr.2020.10956. Epub 2020 Jan 21. PMID: 32016459; PMCID: PMC7002976.
8. Ohnuma K, Kishita Y, Nyuzuki H, Kohda M, Ohtsu Y, Takeo S, Asano T, Sato-Miyata Y, Ohtake A, Murayama K, Okazaki Y, Aigaki T. Ski3/TTC37 deficiency associated with trichohepatoenteric syndrome causes mitochondrial dysfunction in Drosophila. FEBS Lett. 2020 Apr 15. doi: 10.1002/1873-3468.13792. Epub ahead of print. PMID: 32294252.
9. Trojan E, Chamera K, Bryniarska N, Kotarska K, Leśkiewicz M, Regulska M, Basta-Kaim A. Role of Chronic Administration of Antidepressant Drugs in the Prenatal Stress-Evoked Inflammatory Response in the Brain of Adult Offspring Rats: Involvement of the NLRP3 Inflammasome-Related Pathway. Mol Neurobiol. 2019 Aug;56(8):5365-5380. doi: 10.1007/s12035-018-1458-1. Epub 2019 Jan 4. Erratum in: Mol Neurobiol. 2019 Mar 1;: PMID: 30610610; PMCID: PMC6614144.
10. Jeong Nam Y, Kim A, Sung Lee M, Suep Sohn D, Soo Lee C. KATP channel block inhibits the Toll-like receptor 2-mediated stimulation of NF-κB by suppressing the activation of Akt, mTOR, JNK and p38-MAPK. Eur J Pharmacol. 2017 Nov 15;815:190-201. doi: 10.1016/j.ejphar.2017.09.014. Epub 2017 Sep 18. PMID: 28923349.

11. Liu Y, Mao C, Wang M, Liu N, Ouyang L, Liu S, Tang H, Cao Y, Liu S, Wang X, Xiao D, Chen C, Shi Y, Yan Q, Tao Y. Cancer progression is mediated by proline catabolism in non-small cell lung cancer. Oncogene. 2020 Mar;39(11):2358-2376. doi: 10.1038/s41388-019-1151-5. Epub 2020 Jan 7. PMID: 31911619.
12. Mohanraj M, Sekar P, Liou HH, Chang SF, Lin WW. The Mycobacterial Adjuvant Analogue TDB Attenuates Neuroinflammation via Mincle-Independent PLC-γ1/PKC/ERK Signaling and Microglial Polarization. Mol Neurobiol. 2019 Feb;56(2):1167-1187. doi: 10.1007/s12035-018-1135-4. Epub 2018 Jun 6. PMID: 29876879.
13. Wang XP, Luoreng ZM, Zan LS, Li F, Li N. Bovine miR-146a regulates inflammatory cytokines of bovine mammary epithelial cells via targeting the TRAF6 gene. J Dairy Sci. 2017 Sep;100(9):7648-7658. doi: 10.3168/jds.2017-12630. Epub 2017 Jul 6. PMID: 28690061.
14. Yamagishi Y, Someya A, Nagaoka I. Citrulline cooperatively exerts an anti-inflammatory effect on synovial cells with glucosamine and N-acetylglucosamine. Biomed Rep. 2020 Jul;13(1):37-42. doi: 10.3892/br.2020.1304. Epub 2020 May 12. PMID: 32440348; PMCID: PMC7238408.
15. Teke K, Yilmaz H, Uslubas AK, Akpinar G, Kasap M, Mutlu O, Yildiz DK, Guzel N, Dillioglugil O. Histopathologic and molecular comparative analyses of intravesical Aurora kinase-A inhibitor Alisertib with bacillus Calmette-Guérin on precancerous lesions of bladder in a rat model. Int Urol Nephrol. 2018 Aug;50(8):1417-1425. doi: 10.1007/s11255-018-1914-x. Epub 2018 Jun 21. PMID: 29931492.
16. Ning X, Wang Y, Jing M, Sha M, Lv M, Gao P, Zhang R, Huang X, Feng JM, Jiang Z. Apoptotic Caspases Suppress Type I Interferon Production via the Cleavage of cGAS, MAVS, and IRF3. Mol Cell. 2019 Apr 4;74(1):19-31.e7. doi: 10.1016/j.molcel.2019.02.013. Epub 2019 Mar 13. PMID: 30878284.
17. Moon YS, Kwon DR, Lee YJ. Therapeutic effect of microcurrent on calf muscle atrophy in immobilized rabbit. Muscle Nerve. 2018 Aug;58(2):270-276. doi: 10.1002/mus.26110. Epub 2018 Apr 17. PMID: 29466826.
18. Nagappan A, Jung DY, Kim JH, Lee H, Jung MH. Gomisin N Alleviates Ethanol-Induced Liver Injury through Ameliorating Lipid Metabolism and Oxidative Stress. Int J Mol Sci. 2018 Sep 1;19(9):2601. doi: 10.3390/ijms19092601. PMID: 30200508; PMCID: PMC6164513.
19. Tao L, Wu L, Zhang W, Ma WT, Yang GY, Zhang J, Xue DY, Chen B, Liu C. Peroxisome proliferator-activated receptor γ inhibits hepatic stellate cell activation regulated by miR-942 in chronic hepatitis B liver fibrosis. Life Sci. 2020 Jul 15;253:117572. doi: 10.1016/j.lfs.2020.117572. Epub 2020 Mar 19. PMID: 32201276.
20. Feng T, Dzieran J, Yuan X, Dropmann A, Maass T, Teufel A, Marhenke S, Gaiser T, Rückert F, Kleiter I, Kanzler S, Ebert MP, Vogel A, Ten Dijke P, Dooley S, Meindl-Beinker NM. Hepatocyte-specific Smad7 deletion accelerates DEN-induced HCC via activation of STAT3 signaling in mice. Oncogenesis. 2017 Jan 30;6(1):e294. doi: 10.1038/oncsis.2016.85. PMID: 28134936; PMCID: PMC5294248.
21. Seok J, Kim JH, Kim JM, Kwon TR, Choi SY, Li K, Kim BJ. Effects of Intradermal Radiofrequency Treatment and Intense Pulsed Light Therapy in an Acne-induced Rabbit Ear Model. Sci Rep. 2019 Mar 25;9(1):5056. doi: 10.1038/s41598-019-41322-x. PMID: 30911021; PMCID: PMC6434013.

4. Supplementary figure.

Supplementary Figure S1.

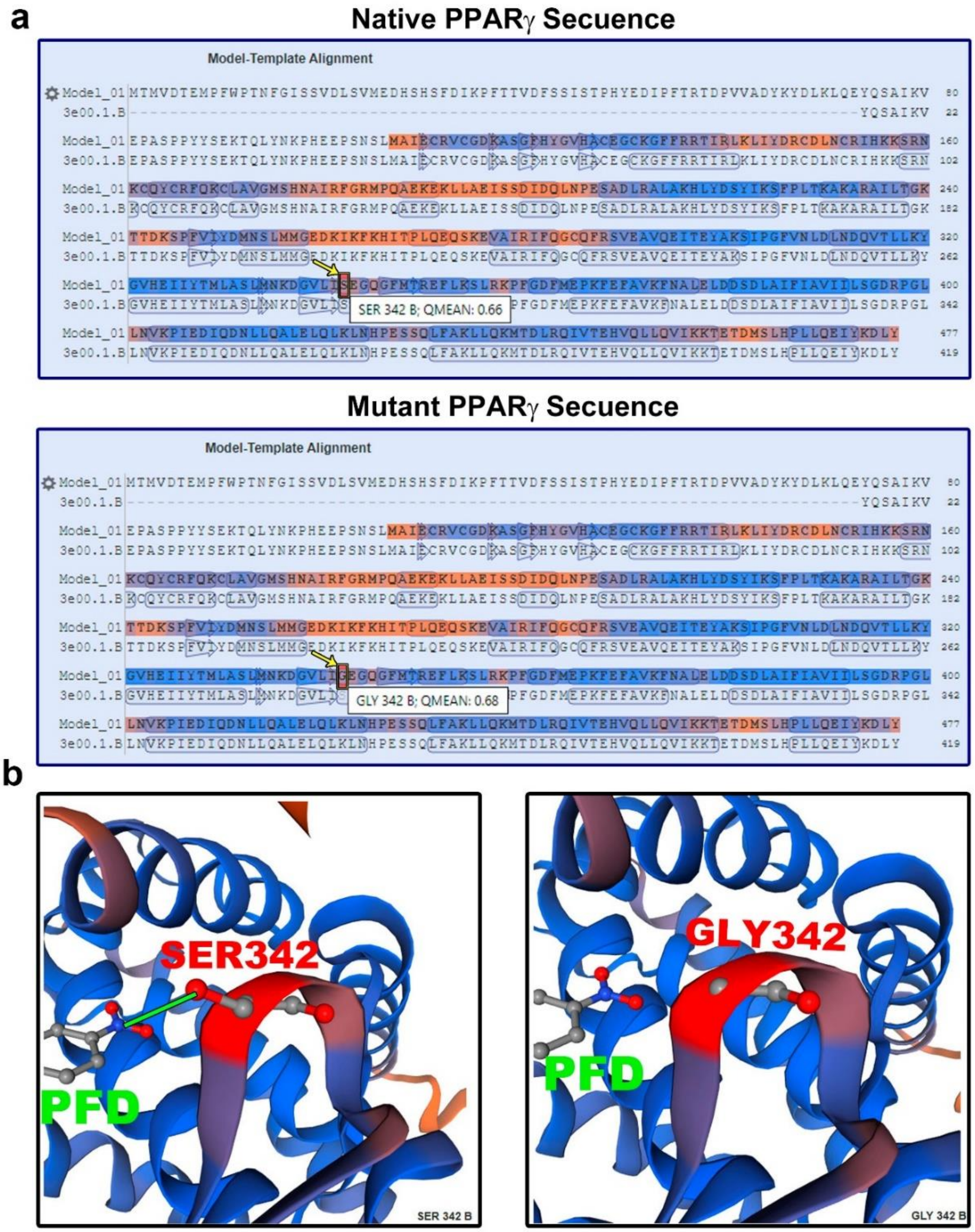

Figure S2. Pirfenidone misses binding affinity to PPARγ mutated in SER342 by GLY342. (**a**) Analysis of PPARγ native and mutant sequence shows that PFD binding is effective when it does not undergo changes in the amino acid sequence. (**b**) Altered amino acid sequence of PPARγ, binding is not favored when Ser is replaced by Gly in the 342 position, which corroborates the agonist role of PFD-PPARγ activation.

5. **Entire blots from Western Blots presented in the figures.**

Dashed lines show where the blots were cute. Samples were run on the same gel but were non-contiguous. Different bands could appear due to re-hybridization process.

Westerns Blots Figure S3C

TGF-beta1

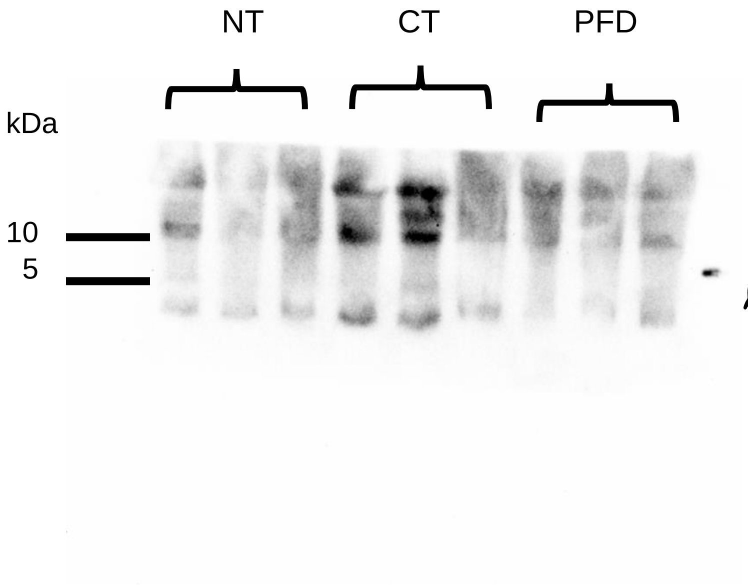

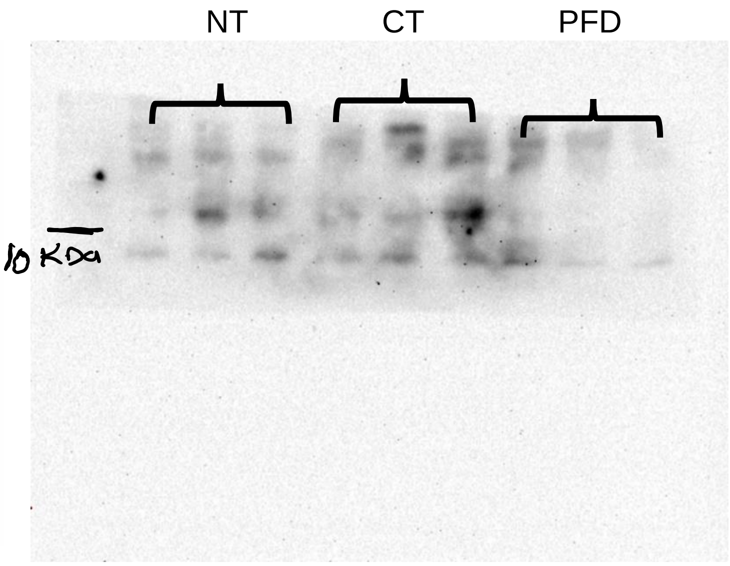

Alpha-SMA

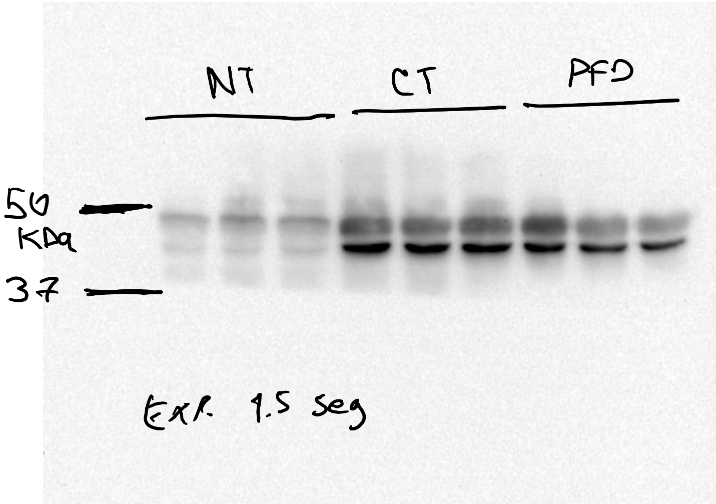

Beta-Tubulin

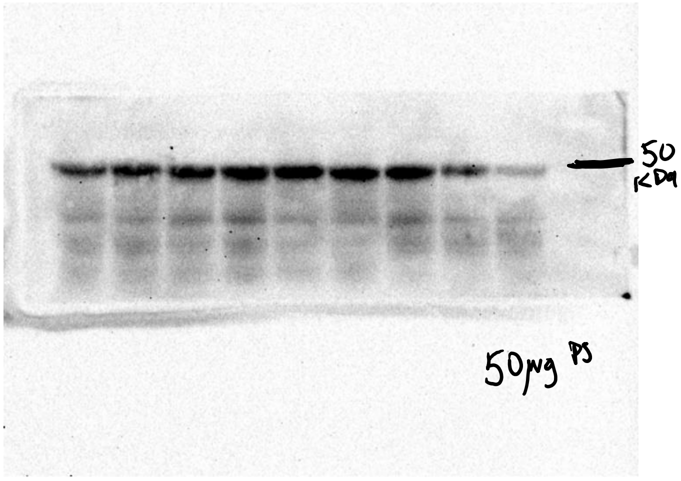

Westerns Blots Figure S4A

IKK-alpha

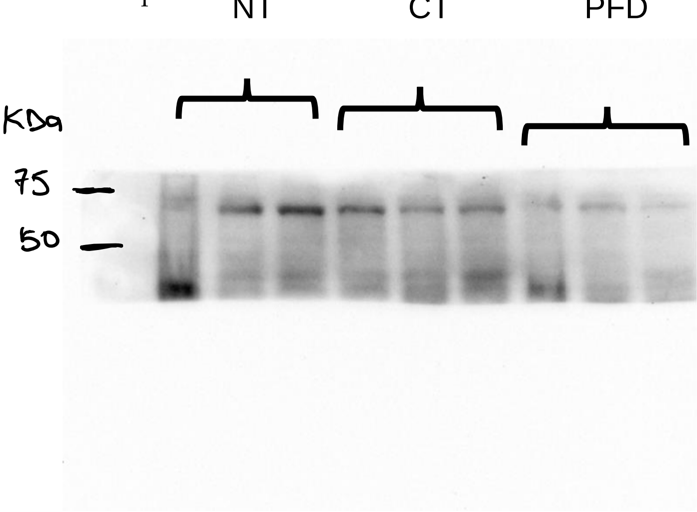

p-IkB-alpha

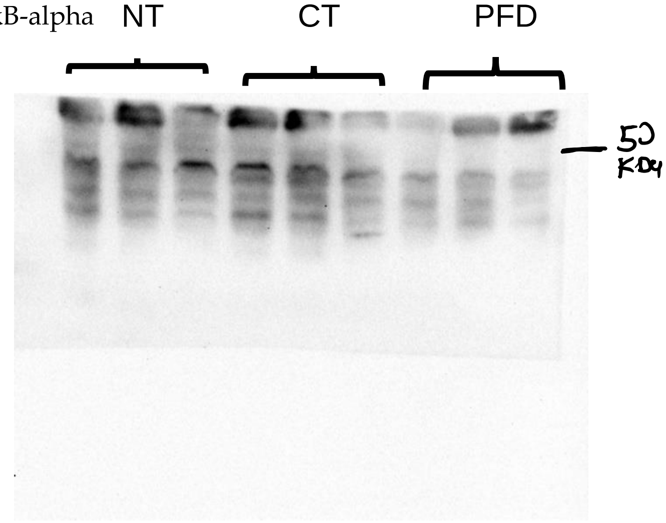

IkB-alpha

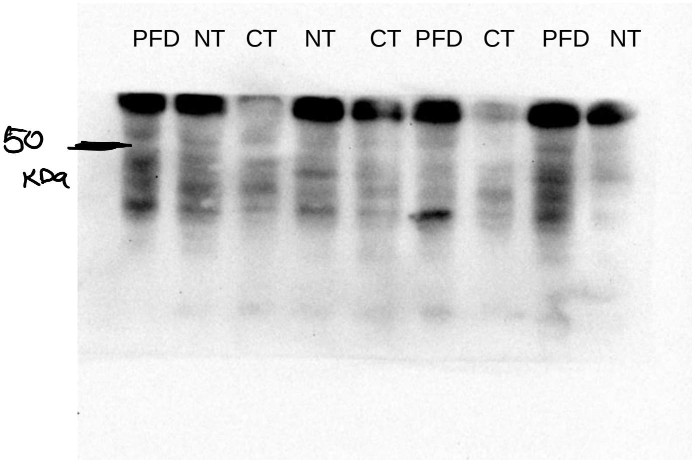

NF-kB p65

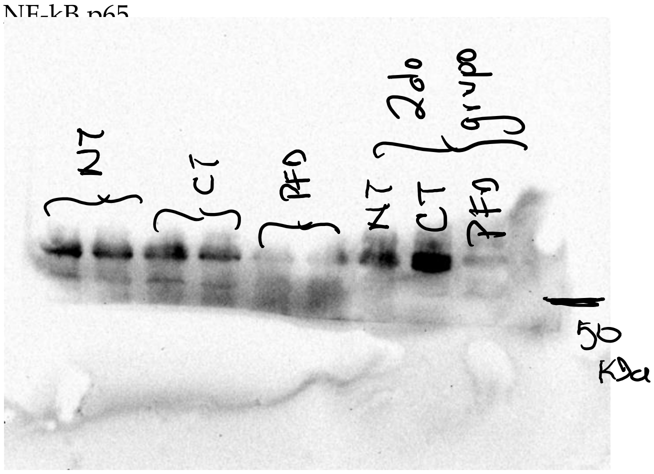

NF-kB p50

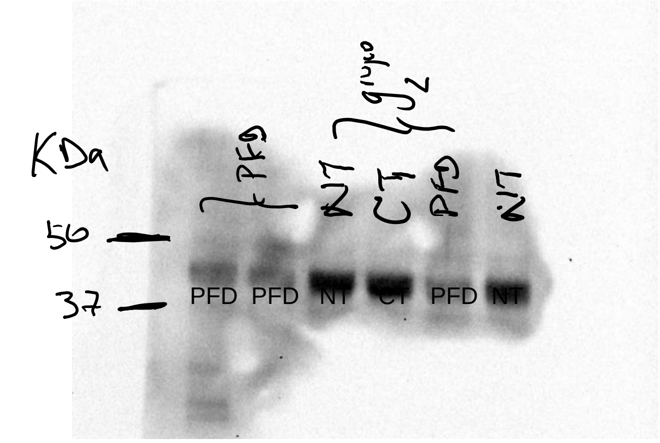

beta-Tubulin

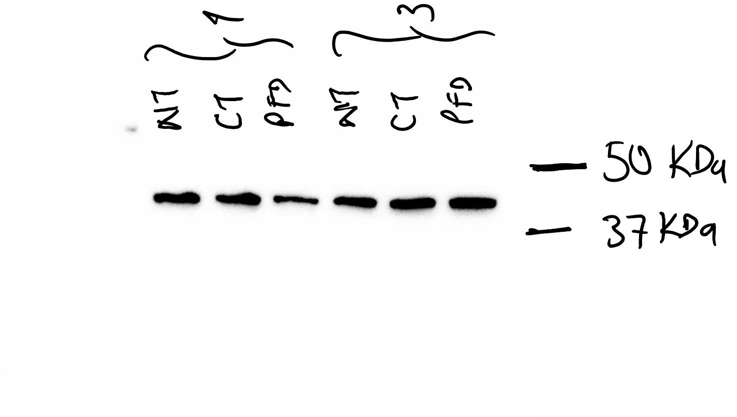

Westerns Blots Figure S4B

NF-kB p65

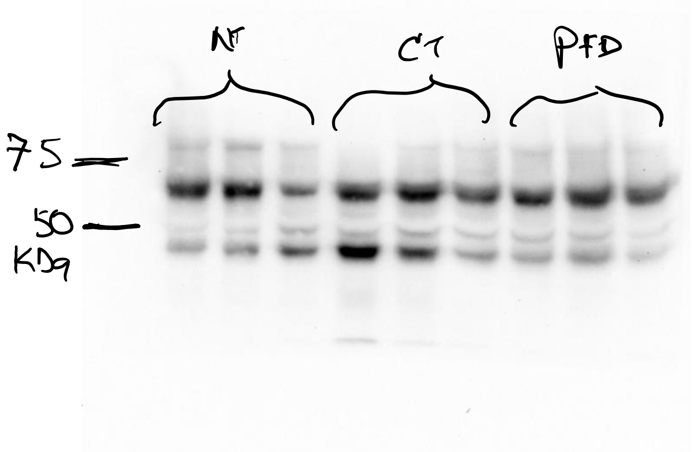

NF-kB p50

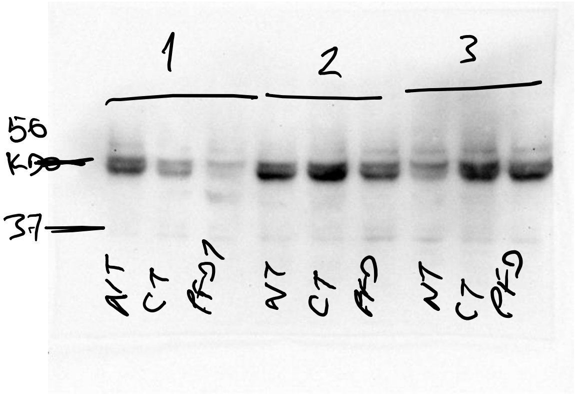

Histone H1

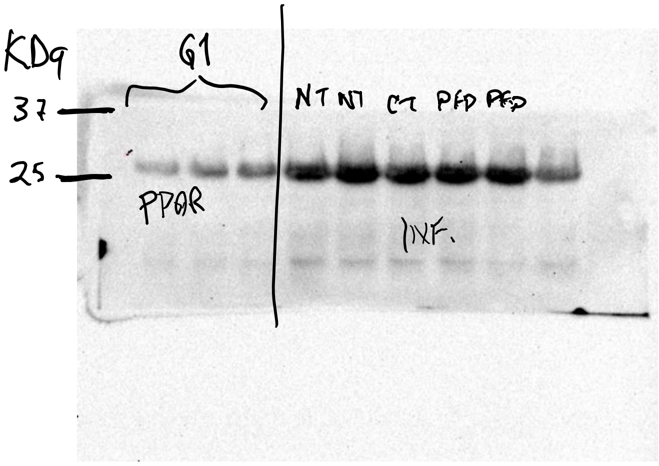

Westerns Blots Figure S4F

IL-6

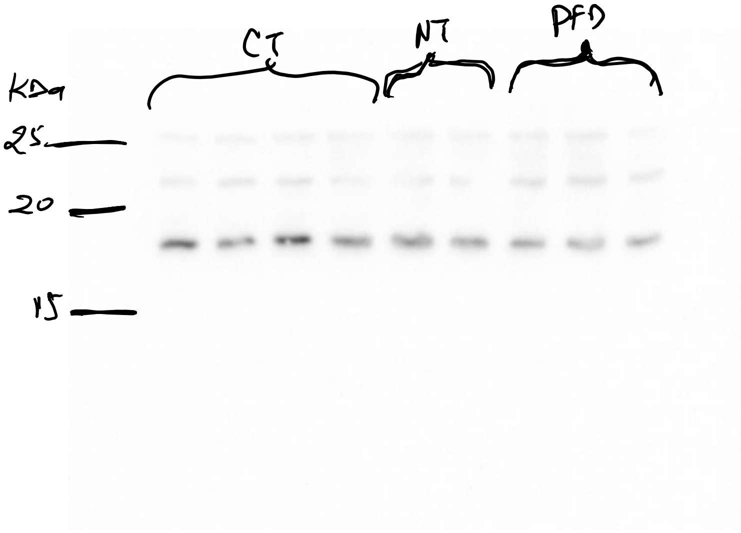

TNF-alpha

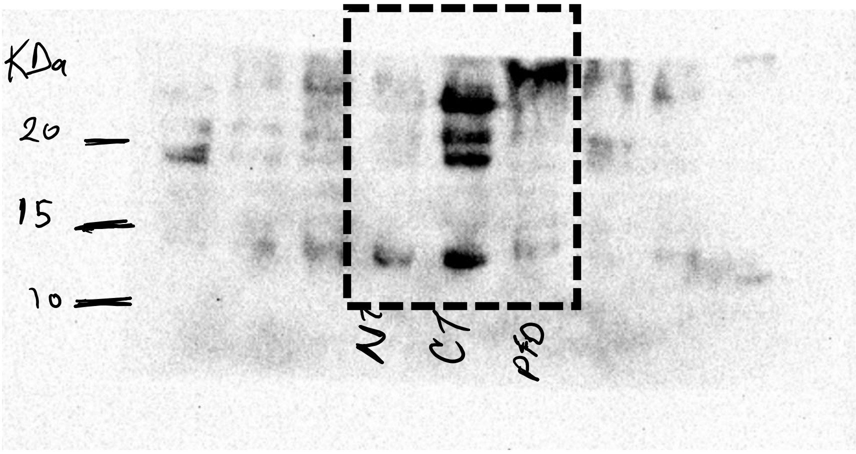

COX-2

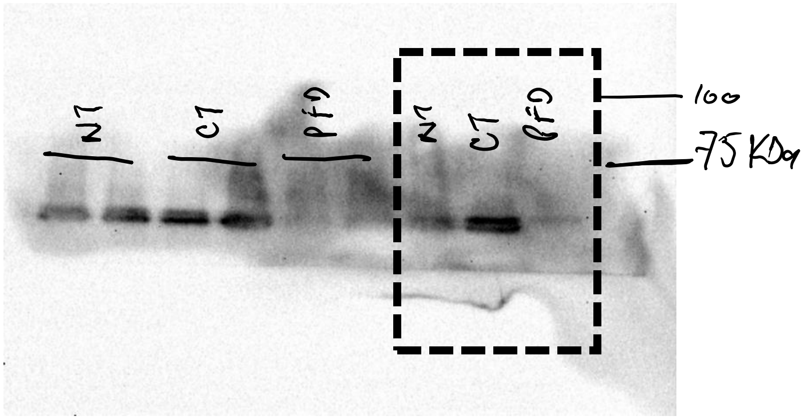

Beta-tubulin

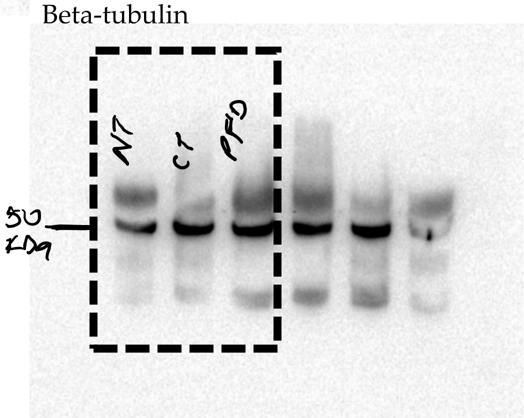

Westerns Blots Figure S5A

PARP-1

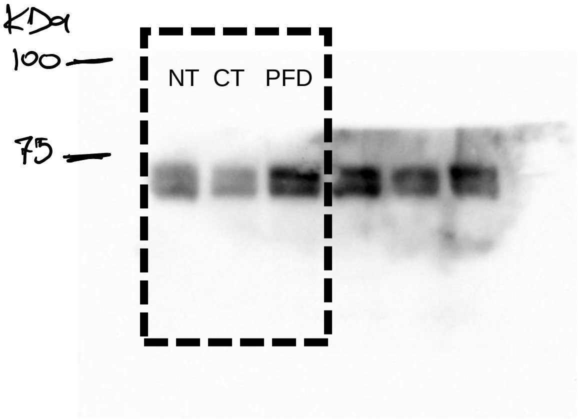

P53

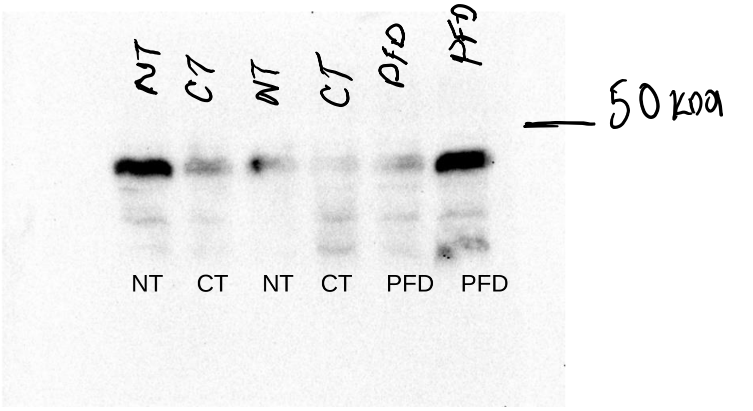

Caspase-3 p17

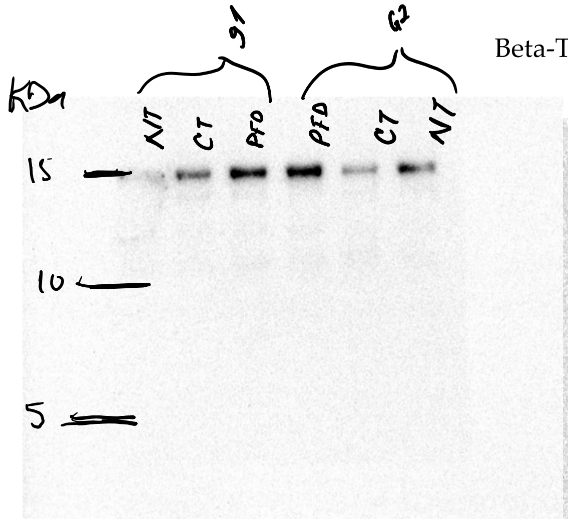

Beta-Tubulin

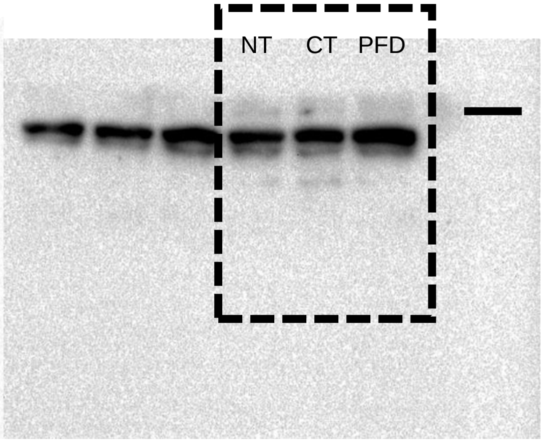

Westerns Blots Figure S5B

PARP-1

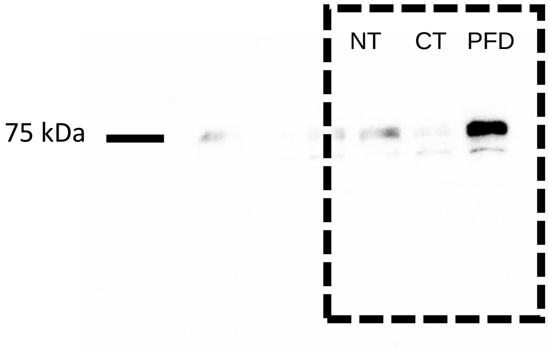

p53

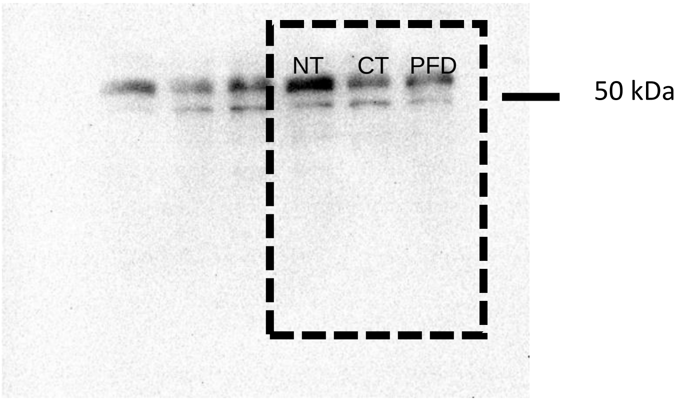

PCNA

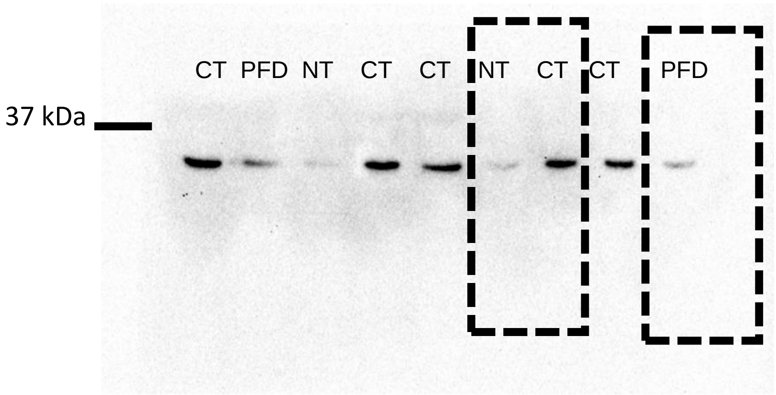

Westerns Blots Figure S6A

PPAR-alpha

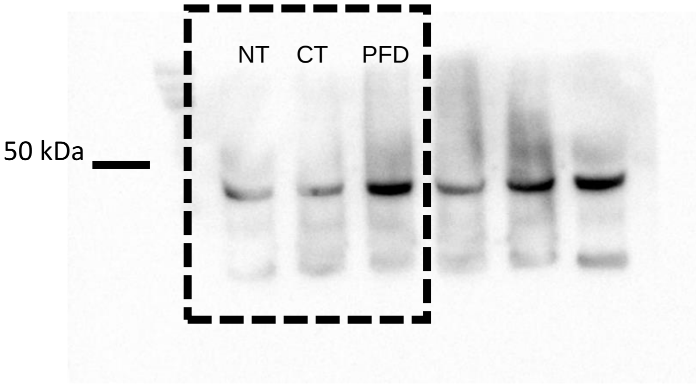

PPAR-gamma

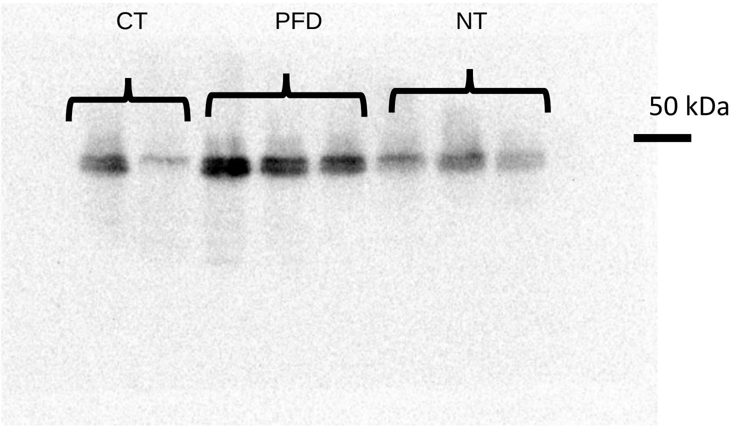

CPT1A

ACOX1

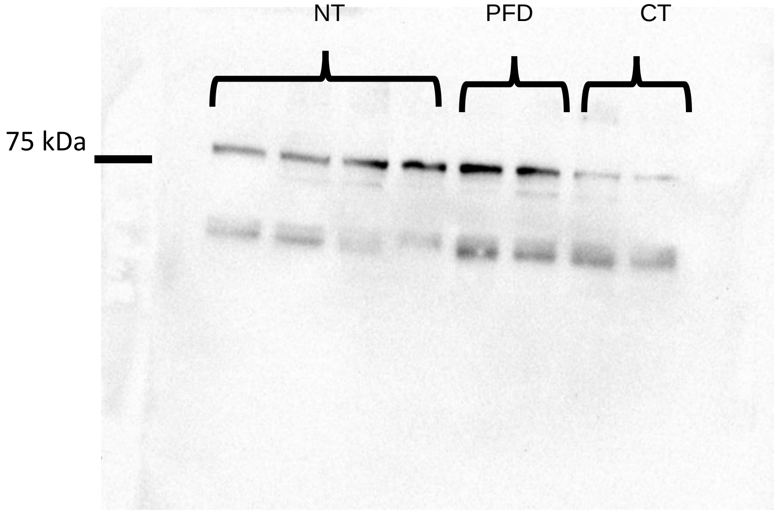

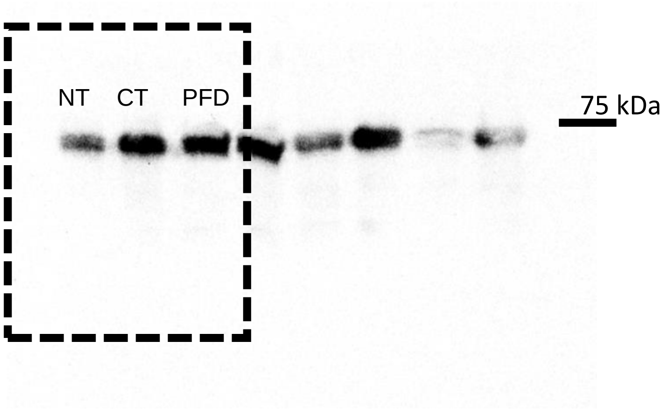

Westerns Blots Figure S6B

PPAR-alpha

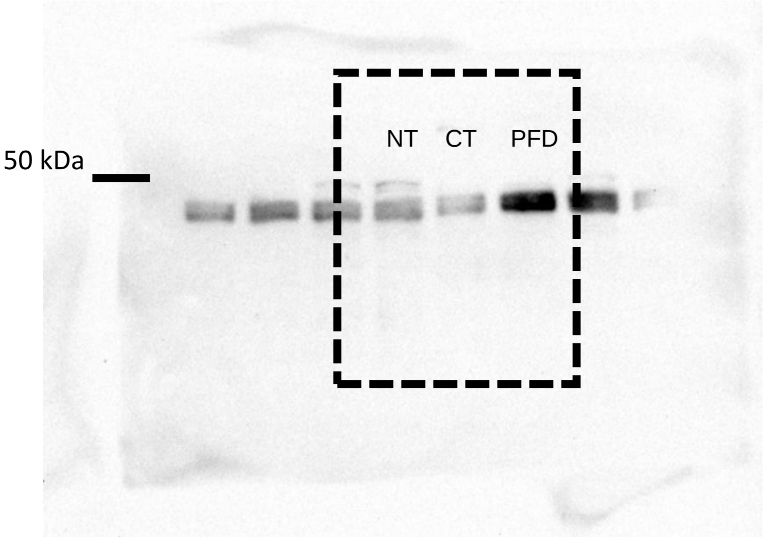

PPAR-gamma

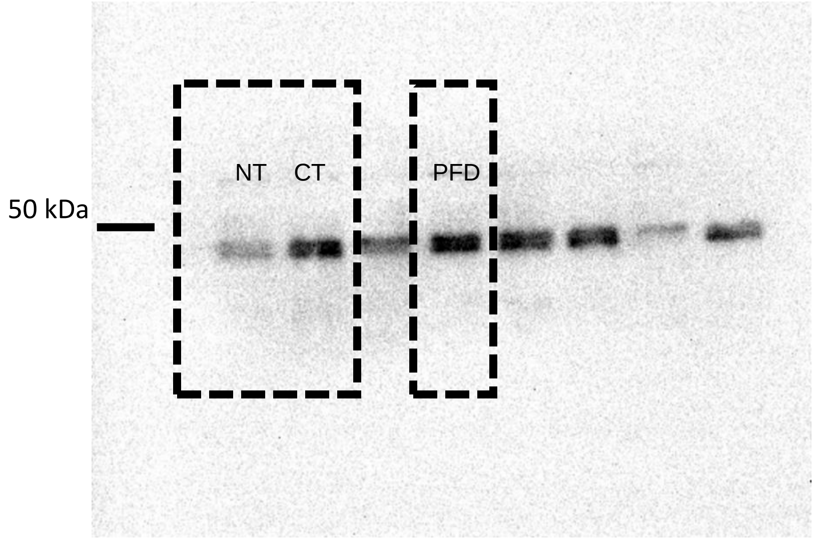

Histone-H1

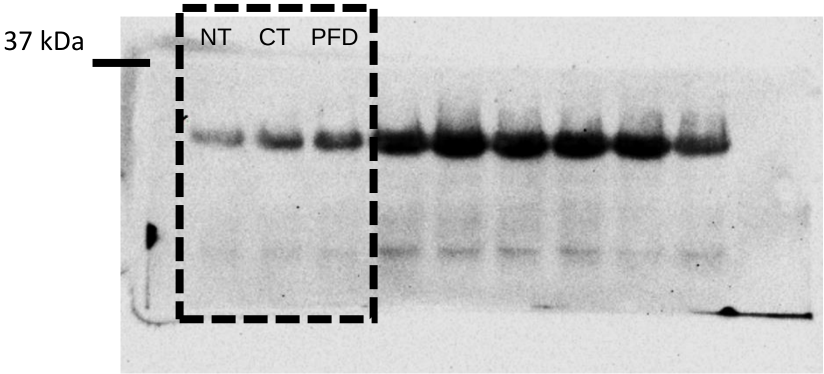

Westerns Blots Figure S6D-E

PPAR-gamma

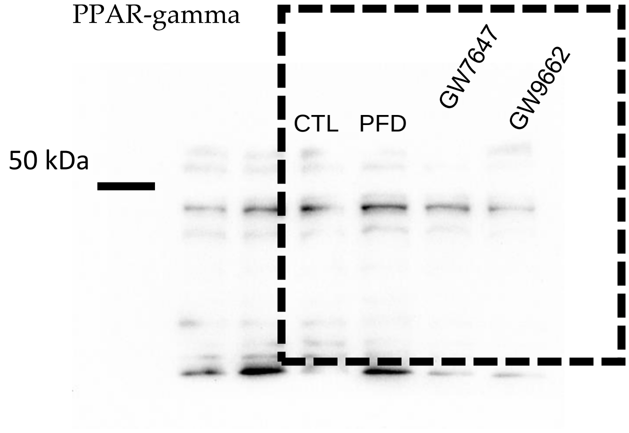

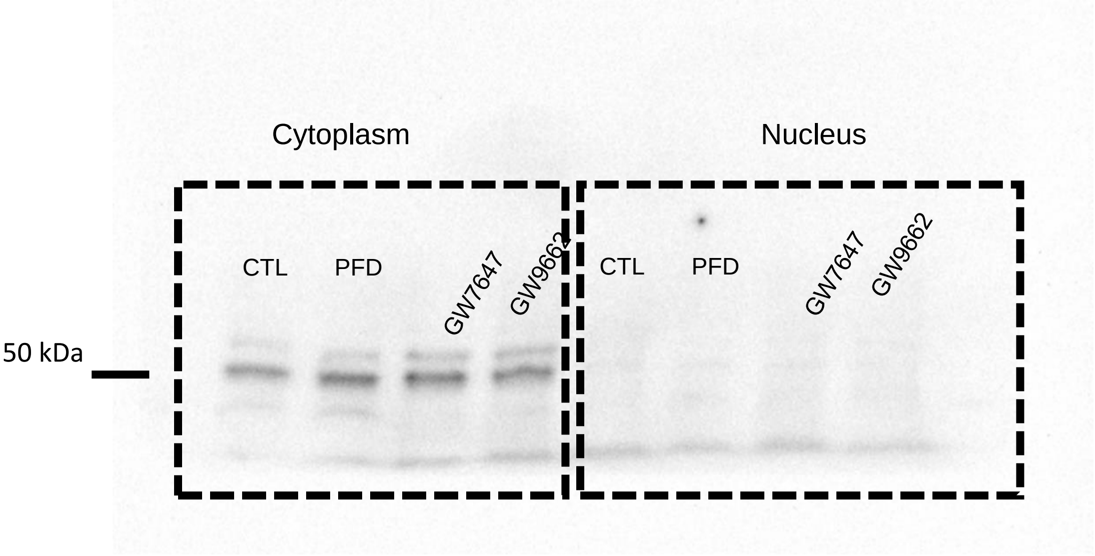

PPAR-alpha cytoplasm Nucleus

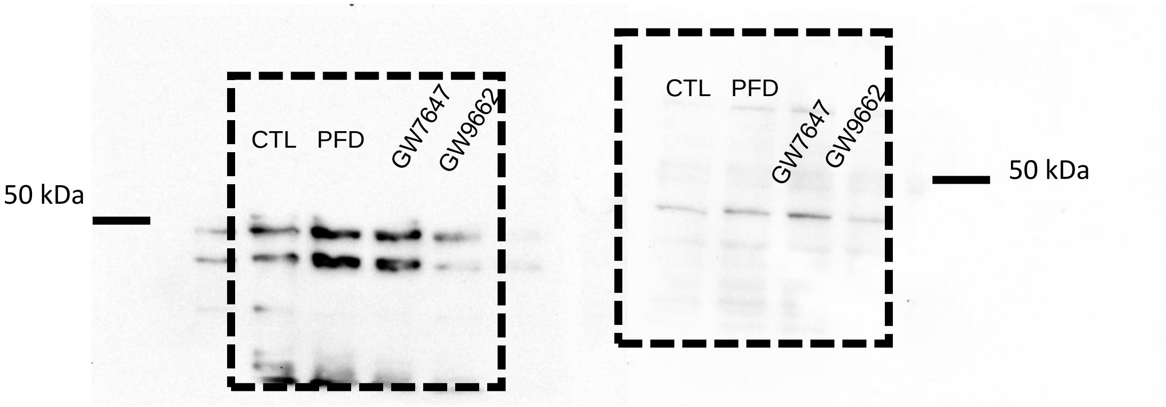

Histone-H3

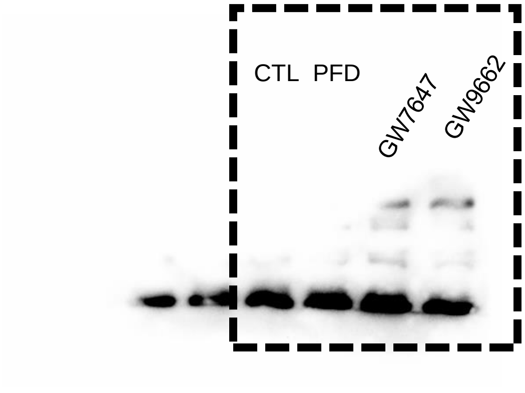

Beta-tubulin

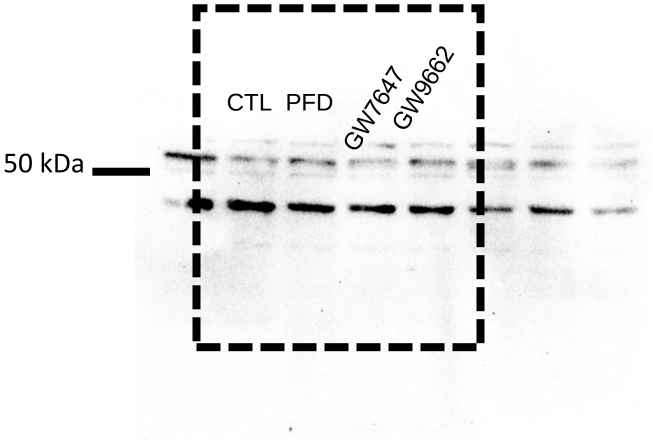

Supplement: Supplementary file 1 [file ijms-22-11360-s001.zip › ijms-1278725-supplementary.pdf]
